# Biosorption of cationic Hg^2+^ and Remazol brilliant blue anionic dye from binary solution using *Gelidium corneum* biomass

**DOI:** 10.1038/s41598-021-00158-0

**Published:** 2021-10-22

**Authors:** Noura El-Ahmady Ali El-Naggar, Ragaa A. Hamouda, Ayman Y. El-Khateeb, Nashwa H. Rabei

**Affiliations:** 1 Department of Bioprocess Development, Genetic Engineering and Biotechnology Research Institute, City of Scientific Research and Technological Applications (SRTA-City), New Borg El-Arab City, Alexandria, 21934 Egypt; 2 Department of Biology, Faculty of Sciences and Arts Khulais, University of Jeddah, Jeddah, Saudi Arabia; 3 Microbial Biotechnology Department, Genetic Engineering and Biotechnology Research Institute, University of Sadat City, Sadat City, Egypt; 4 Department of Agricultural Chemistry, Faculty of Agriculture, Mansoura University, Mansoura, 35516 Egypt

**Keywords:** Environmental sciences, Environmental microbiology

## Abstract

Remazol brilliant blue (RBB) is an anthraquinone anionic dye that has several commercial uses, especially in the textile industries and is well-known for its detrimental impacts on marine life and the surrounding ecosystem. Mercury (Hg^2+^) is also one of the most severe hazardous environmental contaminants due to its bioaccumulation through the food chain and high toxicity to the human embryo and fetus. The biosorption potential of *Gelidium corneum* biomass for bioremoval of Hg^2+^ and RBB dye simultaneously from binary mixture was assessed. The effects of initial pH, contact time, Hg^2+^, RBB, and biomass concentrations on the biosorption process were investigated in 50 batch experiments using a Face-centered central composite design. The maximum removal percentage of Hg^2+^ (98.25%) was achieved in the run no. 14, under optimum experimental conditions: 200 mg/L Hg^2+^, 75 mg/L RBB, pH 5. At 30 °C, 4 g/L algal biomass was used, with a contact time of 180 min. Whereas, the maximum removal percentage of RBB (89.18%) was obtained in the run no. 49 using 200 mg/L Hg^2+^, 100 mg/L RBB, pH 5, 4 g/L algal biomass and 180 min of contact time. FTIR analysis of *Gelidium corneum* biomass surface demonstrated the presence of many functional groups that are important binding sites responsible for Hg^2+^ and RBB biosorption. SEM analysis showed apparent morphological alterations including surface shrinkage and the appearance of new shiny adsorbate ion particles on the *Gelidium corneum* biomass surface after the biosorption process. The EDX study reveals an additional optical absorption peak for Hg^2+^, confirming the role of *Gelidium corneum* biomass in Hg^2+^ biosorption. In conclusion, *Gelidium corneum* biomass has been shown to be an eco-friendly, sustainable, promising, cost-effective and biodegradable biosorbent to simultaneously biosorb Hg^2+^ and RBB dye from aquatic ecosystems.

## Introduction

Heavy metal cations are amongst the most frequent inorganic pollutants in industrial effluents. Environmental pollution by hazardous heavy metals is a serious worldwide concern because of their deposition in the ecosystem; they contaminate food chains and have the potential to cause toxicity and harm to living organisms. Mercury (Hg^2+^) is a hazardous environmental contaminant that is released to the environment from the industrial processes those include fossil fuel burning, batteries and medical devices production, artisanal mining, paint, chlor-alkali, and wood pulp industries^1^. Moreover, mercury is released into the environment, particularly aquatic ecosystems, through the incineration of solid wastes, pyrometallurgical processes (zinc, lead, and iron), coal and oil combustion^2^. Mercury causes massive environmental contamination due to its high toxic effects. It is readily accumulated by living organisms and its subsequent transfer occurs in the food chain through bioaccumulation and bioamplification^3^ and can cause different toxic effects and serious health problems in humans. Mercury has been identified as a high-risk pollutant because it can quickly cross the blood–brain barrier and damage the developing fetal brain^4^. Mercury poisoning by consuming raw seafood causes hydrargyria, a disease that affects the nervous system, lungs, brain, and kidneys^3^. Mercury has a high affinity for protein binding, and it mostly affects the kidney and nervous systems^5^. Elevated concentrations of Hg^2+^ impair lung and kidney functions, as well as induce chest pain and dyspnea^6,7^.

Synthetic dyes are the major industrial coloring molecules widely used in several industries and constitute the main residue found in the textile dyeing industry effluents. It was reported that in some textile dyeing processes, approximately 10–15 percent of the dyes do not bind to the textile fibers and so they are dumped into the wastewater^8^. Per year, about 2.8 × 10^5^ tons of textile dyes are dumped into the wastewater effluents^9^. The dyes affect the water's natural beauty and restrict the penetration of sunlight into the water, which has a detrimental effect on photosynthesis and on aquatic life^10^. Remazol brilliant blue is recalcitrant organic pollutants belongs to the brilliant blue family, is a triphenylmethane dye. It is the most important dye used in textiles and leather industries, electrophoresis, protein quantification, biochemical analysis, etc. It's used in the manufacture of polymeric dyes as a starting material^11^. The discharge of Remazol brilliant blue dye into the water bodies causes a serious environmental risk due to its biodegradation resistant, highly toxic, highly visible due to its bright color in effluent “even at a very low concentration of less than 1 ppm”, harmful to aquatic life, carcinogenic and mutagenic properties^12^. Remazol brilliant blue and its breakdown products have potential health effects including respiratory and digestive tract irritation, hyperactivity in children, different types of allergies, eye and skin irritation, asthma, migraines, etc.^12^.

Several reports on single-pollutant adsorption have been conducted, even though industrial effluents contain significant concentrations of both dyes and metals^13^. Therefore, attention must be paid to the simultaneous removal of dyes and heavy metal ions during wastewater treatment processes^14^. Several traditional methods were used to remove the water pollutants, including chemical oxidation or reduction, chemical precipitation, evaporation and adsorption, filtration, reverse osmosis, electrochemical treatment and ion exchange^15^. Nevertheless, these approaches are often limited in terms of technological feasibility due to a high cost, ineffectiveness in achieving legal discharge levels (especially in low concentrations effluents), the use of a lot of reagents or energy, as well as the production of a lot of toxic wastes.

Consequently, it is urgent to find cost-effective alternative technologies for simultaneous removal of the contaminants during wastewater treatment processes. The biosorption process is an efficient alternative biological treatment technique that utilize the biosorbents to eliminate or degrade harmful pollutants that are hazardous to public health and/or the ecosystems into less toxic compounds with concentration ranges below regulatory authorities' concentration limits. A variety of natural biosorbents have been used to separate the contaminants such as grape shoot, olive core, peat^16^. Microorganisms as fungi^17^, bacteria^18^ and several marine macro algal biomasses were used to remediate the contaminants from wastewater^19^. Several studies have shown that removal of the contaminants from wastewater by microorganisms has many advantages over conventional treatment methods. These include high efficacy in diluted effluents, environmental friendliness, high metal binding ability, utilizing different cost-effective biosorbents, minimization of chemical and/or biological sludge, and the ability to regenerate biosorbents with the possibility of recovering the metal^20^.

The algae are efficient and are known to be cheap renewable biosorbents as the nutrition requirements by algae are low, don’t produce toxic substances and possess a high metal binding capacity^21^. Due to the presence of lipids, polysaccharides or proteins on their surface, algae biomasses have high metal binding capacities^21–23^. Dulla et al.^24^ documented that the biosorption capability of algae is mainly based on the presence of various functional groups of polysaccharides, proteins and lipids in algae cell walls including amino, carboxylic, hydroxyl and sulfate groups, which serve as adsorption sites for metal ions through a variety of mechanisms including ion exchange, electrostatic forces, and complexation. El-Naggar et al.^25,26^ and Hamouda et al.^27^ were able to successfully remove textile dyes and heavy metals using various types of algal biomass. Dead algal biomass has proven to be more appropriate as biosorbents than living cells because it requires no nutrients, can be stored and used for extended periods of time, and can be recovered with organic solvents or surfactants^28^. Several adsorbents were investigated for the decontamination of hazardous wastewater pollutants such as polyurethane-based adsorbents, polysulfone-immobilized *Turbinaria conoides* biomass, nano-based products like nanocomposite, nano adsorbent^29–33^.

The main objective of this research was to assess the capacity of *Gelidium corneum* biomass for the simultaneous biosorption of Hg^2+^ and Remazol brilliant blue dye from binary solution, to optimize the impacts of the process factors on Remazol brilliant blue dye and Hg^2+^ removal percentages using response surface methodology and to characterize the *Gelidium corneum* biomass pre and post the biosorption process.

## Results and discussion

In the current study, the red alga *Gelidium corneum* biomass was used as a sustainable feedstock for the biosorption process of Hg^2+^ and Remazol brilliant blue dye from binary solution. The cell walls of red algae are made up of a backbone of cellulose, xylan, and mannan fibrils, as well as a huge matrix of polysaccharides such as agar, carrageen, gelans, and protein, as well as minerals. Calcium carbonate may be deposited in the cell walls of some red seawater algae^34^. The cell wall components of the red algae, in addition to calcium carbonate, can provide a range of different binding sites on the surface of the algal biomass that are responsible for the metal ions and dye biosorption.

### Statistical optimization of simultaneous biosorption process of Hg^2+^ and Remazol brilliant blue dye by *Gelidium corneum* biomass

The simultaneous biosorption process of dyes and heavy metals by algae could be affected by many operating process variables such as contact time, initial pH level, algal biomass dosage, initial metal and dye concentrations^25,26^. It is very important to optimize the operating process variables for the simultaneous biosorption process. Statistical methods such as Response surface methodology (RSM) which is a well-used optimization method, can be used to optimize operational variables in order to maximize a specific response. RSM is a set of mathematical and statistical techniques useful for the modelling and analysis of data in which one or more response variables of interest are influenced by several variables and the objective is to optimize these responses It is commonly used to explain the relationships between the different processing parameters and to identify the optimum values of the variables that have a significant impact on the response. The main concept of RSM is to obtain an optimal response through a series of carefully designed experiments. The goals for RSM are to understand how the response changes in a given direction by adjusting the design variables, to maintain a high efficiency with respect to economical cost, time, and any other practical limitations, to predict the observed response as accurately and precisely as possible in areas of the experimental region where no experiments have been performed. Propose successive methods for conducting experiments with various variables, to estimate the experimental variance with confidence (pure error)^25,26,35^.

**Figure 1:**
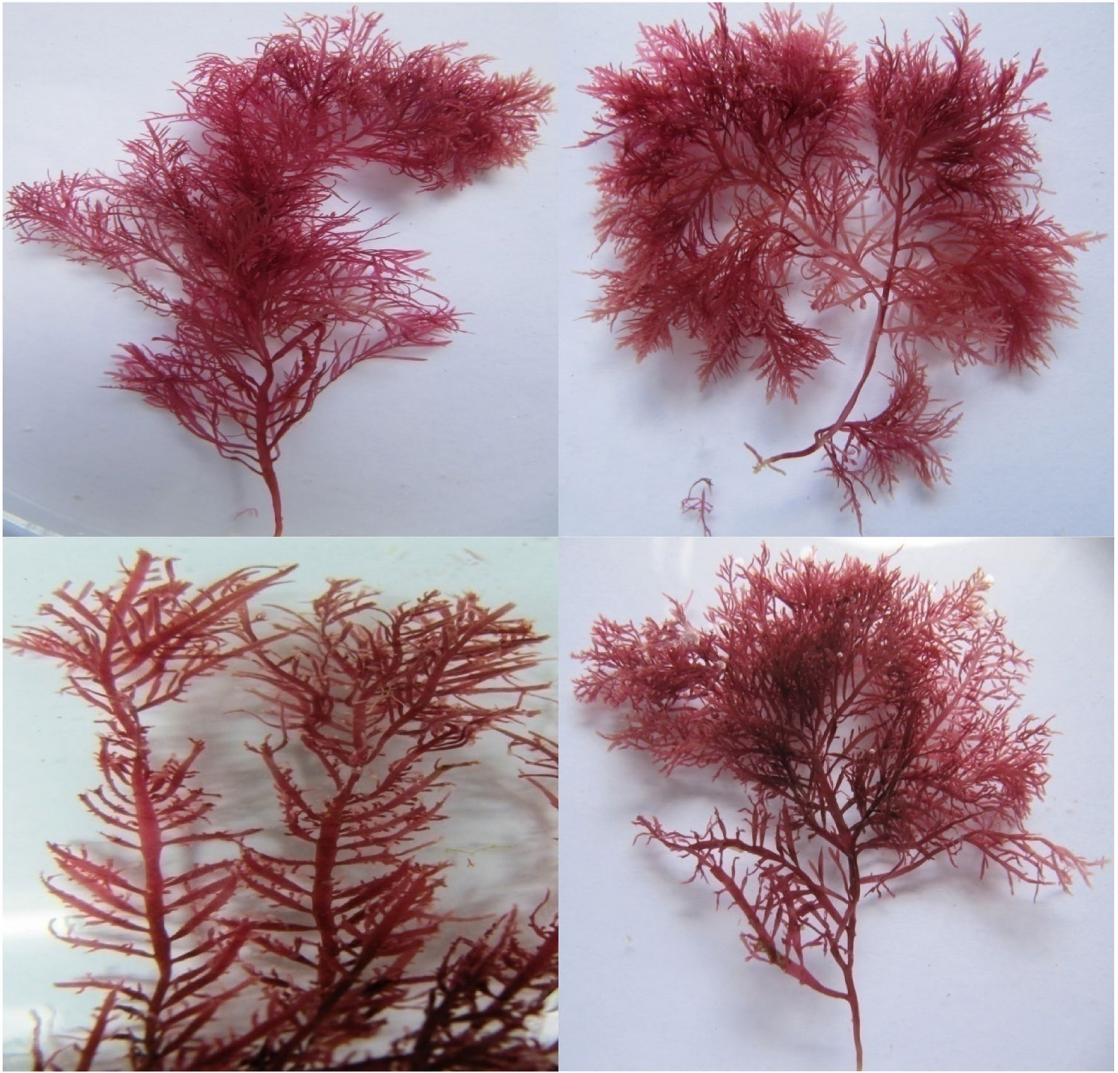
*Gelidium corneum*.

Optimization of the biosorption process of Hg^2+^ and Remazol brilliant blue dye has been achieved in batch experiments to improve the bioremoval percentages. Figure 1 showed the collected biomass of *Gelidium corneum* used for the biosorption process. Table 1 displays the FCCCD with eight center-points, 10 axial points, and 32 factorial points. The actual and coded levels of the five independent factors, as well as the experimental and predicted Hg^2+^ and Remazol brilliant blue dye removal percentages and the residuals, also displayed in Table 1. The findings obtained from FCCCD experiments for Hg^2+^ and Remazol brilliant blue dye biosorption indicate substantial variations in the percentages of removal of Hg^2+^ and Remazol brilliant blue dye. The removal percentages of Hg^2+^ ranged from 62.70 to 98.25 percent, while the Remazol brilliant blue dye removal percentages varied from 48.75 to 89.18 percent. The maximum removal percentage of Hg^2+^ with a percent of 98.25% was achieved in the run no. 14 at 30 °C under the optimum experimental conditions: 200 mg/L Hg^2+^, 75 mg/L Remazol brilliant blue dye, initial pH 5, 4 g/L *Gelidium corneum* biomass and 180 min of contact time. Whereas, the maximum removal percentage of Remazol brilliant blue dye with percent of 89.18 was achieved in the run no. 49 at 30 °C, under optimum experimental conditions: 200 mg/L Hg^2+^, 100 mg/L Remazol brilliant blue dye, initial pH 5, 4 g/L *Gelidium corneum* biomass and 180 min of contact time. In addition, the lowest removal percentages of Hg^2+^ and Remazol brilliant blue dye were recorded in the experimental runs no. 48 and 9; respectively. The biosorption process capacity was reduced in the run no. 48, which could be attributed to low levels of Hg^2+^ concentration, *Gelidium corneum* biomass concentration, contact time and initial pH.

**Table 1:** FCCCD matrix mean actual and predicted values of simultaneous biosorption of Hg^2+^ and Remazol brilliant blue dye by using *Gelidium corneum* biomass.

Std	Run	Type	X_1_	X_2_	X_3_	X_4_	X_5_	Remazol brilliant blue removal (%)			Hg^2+^ removal (%)		
								Actual	Predicted	Residuals	Actual	Predicted	Residuals
41	1	Axial	0	0	0	0	− 1	78.60	79.29	− 0.69	93.07	94.30	− 1.23
32	2	Fact	1	1	1	1	1	70.36	68.77	1.60	78.95	78.48	0.47
7	3	Fact	− 1	1	1	− 1	− 1	71.74	71.13	0.61	77.85	77.59	0.26
33	4	Axial	− 1	0	0	0	0	74.91	75.84	− 0.93	87.61	88.52	− 0.91
9	5	Fact	− 1	− 1	− 1	1	− 1	58.96	58.85	0.11	70.10	70.20	− 0.11
23	6	Fact	− 1	1	1	− 1	1	68.81	68.40	0.41	76.13	75.74	0.39
13	7	Fact	− 1	− 1	1	1	− 1	58.19	58.58	− 0.40	74.55	74.08	0.47
49	8	Center	0	0	0	0	0	82.29	82.89	− 0.60	95.59	96.44	− 0.85
2	9	Fact	1	− 1	− 1	− 1	− 1	48.75	49.75	− 1.00	68.04	67.89	0.15
43	10	Center	0	0	0	0	0	82.57	82.89	− 0.31	97.59	96.44	1.15
25	11	Fact	− 1	− 1	− 1	1	1	66.15	65.75	0.39	79.90	79.91	− 0.01
29	12	Fact	− 1	− 1	1	1	1	56.19	55.64	0.55	74.53	74.84	− 0.31
30	13	Fact	1	− 1	1	1	1	58.57	57.77	0.80	72.75	72.28	0.47
46	14	Center	0	0	0	0	0	81.46	82.89	− 1.43	98.25	96.44	1.81
45	15	Center	0	0	0	0	0	84.61	82.89	1.72	97.31	96.44	0.87
20	16	Fact	1	1	− 1	− 1	1	75.91	74.99	0.92	90.13	90.47	− 0.34
14	17	Fact	1	− 1	1	1	− 1	57.19	57.59	− 0.40	76.04	76.53	− 0.49
28	18	Fact	1	1	− 1	1	1	74.91	76.39	− 1.48	86.33	86.34	− 0.02
4	19	Fact	1	1	− 1	− 1	− 1	63.85	64.73	− 0.88	88.86	88.39	0.47
37	20	Axial	0	0	− 1	0	0	75.73	76.49	− 0.76	94.30	95.89	− 1.59
47	21	Center	0	0	0	0	0	83.18	82.89	0.29	98.09	96.44	1.65
34	22	Axial	1	0	0	0	0	77.93	77.39	0.54	91.00	91.88	− 0.87
21	23	Fact	− 1	− 1	1	− 1	1	60.04	60.71	− 0.67	72.16	72.19	− 0.04
42	24	Axial	0	0	0	0	1	83.24	82.94	0.30	95.16	95.72	− 0.55
40	25	Axial	0	0	0	1	0	80.51	80.50	0.01	94.58	95.52	− 0.93
8	26	Fact	1	1	1	− 1	− 1	73.24	72.25	1.00	88.22	88.08	0.15
19	27	Fact	− 1	1	− 1	− 1	1	71.03	70.89	0.14	86.73	86.19	0.54
50	28	Center	0	0	0	0	0	80.96	82.89	− 1.93	96.31	96.44	− 0.13
27	29	Fact	− 1	1	− 1	1	1	71.80	71.35	0.44	86.76	86.19	0.57
44	30	Center	0	0	0	0	0	84.62	82.89	1.73	98.22	96.44	1.79
39	31	Axial	0	0	0	− 1	0	78.54	78.94	− 0.40	92.19	93.04	− 0.85
31	32	Fact	− 1	1	1	1	1	62.77	63.57	− 0.81	76.48	77.13	− 0.65
10	33	Fact	1	− 1	− 1	1	− 1	58.61	57.70	0.90	71.32	71.45	− 0.13
35	34	Axial	0	− 1	0	0	0	76.87	77.49	− 0.62	85.32	84.60	0.72
12	35	Fact	1	1	− 1	1	− 1	74.52	72.93	1.59	91.26	90.69	0.57
17	36	Fact	− 1	− 1	− 1	− 1	1	65.29	65.54	− 0.25	78.45	78.65	− 0.20
38	37	Axial	0	0	1	0	0	75.56	75.19	0.37	93.00	93.20	− 0.19
18	38	Fact	1	− 1	− 1	− 1	1	68.29	66.58	1.71	79.33	79.01	0.32
5	39	Fact	− 1	− 1	1	− 1	− 1	57.33	56.86	0.47	64.65	65.00	− 0.35
26	40	Fact	1	− 1	− 1	1	1	66.00	67.74	− 1.74	75.91	76.14	− 0.23
15	41	Fact	− 1	1	1	1	− 1	73.11	73.10	0.01	85.75	85.40	0.34
11	42	Fact	− 1	1	− 1	1	− 1	71.09	71.02	0.07	85.69	85.53	0.17
24	43	Fact	1	1	1	− 1	1	71.06	72.65	− 1.59	81.34	81.21	0.13
22	44	Fact	1	− 1	1	− 1	1	61.17	61.90	− 0.73	73.22	73.75	− 0.53
16	45	Fact	1	1	1	1	− 1	73.52	75.16	− 1.63	91.59	91.77	− 0.18
48	46	Center	0	0	0	0	0	84.99	82.89	2.10	97.26	96.44	0.82
3	47	Fact	− 1	1	− 1	− 1	− 1	63.14	63.76	− 0.63	78.74	79.10	− 0.35
1	48	Fact	− 1	− 1	− 1	− 1	− 1	52.30	51.83	0.47	62.70	62.51	0.19
36	49	Axial	0	1	0	0	0	89.18	88.95	0.23	93.49	95.99	− 2.50
6	50	Fact	1	− 1	1	− 1	− 1	55.33	54.93	0.41	71.65	71.58	0.07

Variable	Code	− 1	0	1									
Hg^2+^ concentration (mg/L)	X_1_	100	200	300									
Remazol brilliant blue concentration (mg/L)	X_2_	50	75	100									
Initial pH level	X_3_	3	5	7									
Contact time (min)	X_4_	60	180	300									
Algal biomass concentration (g/L)	X_5_	1	4	7									

### Multiple regression analysis and ANOVA

The multiple regression method was used to analyze the results of Hg^2+^ and Remazol brilliant blue dye removal (%), and the findings were provided in Tables 2, 3, 4 and 5. The regression analysis involves the adjusted R^2^ value, the predicted R^2^ value, the coefficient of determination (R^2^), Fisher test (*F*-test) and the probability *P *value. The chosen variables' linear, interaction, and quadratic effects have also been assessed. The negative coefficient values of linear effects, quadratic effects and interactions of the chosen process variables indicate that they have a negative effect on Hg^2+^ and Remazol brilliant blue dye removal percentages by *Gelidium corneum* biomass, while the positive coefficient values imply an increase in Hg^2+^ and Remazol brilliant blue dye removal percentages by *Gelidium corneum* biomass in the tested values of the chosen variables. It's obvious from the coefficient values (Tables 2, 3) that the concentrations of Hg^2+^, Remazol brilliant blue and algal biomass, contact time had positive impacts on both Remazol brilliant blue dye and Hg^2+^ removal percentages by *Gelidium corneum* biomass. However, the initial pH level had a negative impact on both Remazol brilliant blue dye and Hg^2+^ removal percentages by *Gelidium corneum* biomass.

**Table 2:** Analysis of variance for biosorption of Remazol brilliant blue dye by using *Gelidium corneum* biomass obtained by FCCCD.

Source of variance	Degrees of freedom	Sum of square	Mean of square	*F* value	*P* value	Coefficient estimate
Model	20	4823.47	241.17	147.43	< 0.0001*	82.89
**Linear effect**						
X_1_	1	20.50	20.50	12.53	0.0014*	0.78
X_2_	1	1116.42	1116.42	682.47	< 0.0001*	5.73
X_3_	1	14.37	14.37	8.79	0.0060*	− 0.65
X_4_	1	20.80	20.80	12.72	0.0013*	0.78
X_5_	1	113.58	113.58	69.43	< 0.0001*	1.83
**Interaction effect**						
X_1_X_2_	1	18.66	18.66	11.41	0.0021*	0.76
X_1_X_3_	1	0.05	0.05	0.03	0.8681	0.04
X_1_X_4_	1	1.78	1.78	1.09	0.3058	0.24
X_1_X_5_	1	19.57	19.57	11.96	0.0017*	0.78
X_2_X_3_	1	10.92	10.92	6.68	0.0151*	0.58
X_2_X_4_	1	0.12	0.12	0.07	0.7888	0.06
X_2_X_5_	1	86.42	86.42	52.83	< 0.0001*	− 1.64
X_3_X_4_	1	55.95	55.95	34.20	< 0.0001*	− 1.32
X_3_X_5_	1	194.25	194.25	118.75	< 0.0001*	− 2.46
X_4_X_5_	1	92.39	92.39	56.48	< 0.0001*	− 1.70
**Quadratic effect**						
X_1_^2^	1	97.27	97.27	59.46	< 0.0001*	− 6.27
X_2_^2^	1	0.28	0.28	0.17	0.6822	0.34
X_3_^2^	1	122.85	122.85	75.10	< 0.0001*	− 7.05
X_4_^2^	1	24.82	24.82	15.17	0.0005*	− 3.17
X_5_^2^	1	7.79	7.79	4.76	0.0374*	− 1.77
**Error effect**						
Lack of Fit	22	31.07	1.41	0.60	0.8276	82.89
Pure error	7	16.36	2.34			0.78

R^2^	0.9903	SD	1.28			
Adj R^2^	0.9835	Mean	70.70			
Pred R^2^	0.9718	C.V. %	1.81			
Adeq precision	47.30	PRESS	137.12			

*F* Fishers’s function, *P* level of significance, *C.V* coefficient of variation.

*Significant values.

**Table 3:** Analysis of variance for biosorption of Hg^2+^ by *Gelidium corneum* biomass obtained by FCCCD.

Source of variance	Degrees of freedom	Sum of square	Mean of square	*F* value	*P* value	Coefficient estimate
Model	20	4901.17	245.06	229.45	< 0.0001*	96.44
**Linear effect**						
X_1_	1	96.14	96.14	90.01	< 0.0001*	1.68
X_2_	1	1103.34	1103.34	1033.07	< 0.0001*	5.70
X_3_	1	61.47	61.47	57.55	< 0.0001*	− 1.34
X_4_	1	52.13	52.13	48.81	< 0.0001*	1.24
X_5_	1	17.17	17.17	16.08	0.0004*	0.71
**Interaction effect**						
X_1_X_2_	1	30.67	30.67	28.72	< 0.0001*	0.98
X_1_X_3_	1	2.87	2.87	2.69	0.1120	0.30
X_1_X_4_	1	34.03	34.03	31.86	< 0.0001*	− 1.03
X_1_X_5_	1	50.35	50.35	47.14	< 0.0001*	− 1.25
X_2_X_3_	1	31.98	31.98	29.94	< 0.0001*	− 1.00
X_2_X_4_	1	3.18	3.18	2.98	0.0949	− 0.32
X_2_X_5_	1	163.42	163.42	153.01	< 0.0001*	− 2.26
X_3_X_4_	1	3.86	3.86	3.61	0.0673	0.35
X_3_X_5_	1	160.03	160.03	149.84	< 0.0001*	− 2.24
X_4_X_5_	1	82.66	82.66	77.40	< 0.0001*	− 1.61
**Quadratic effect**						
X_1_^2^	1	96.29	96.29	90.16	< 0.0001*	− 6.24
X_2_^2^	1	93.28	93.28	87.34	< 0.0001*	− 6.14
X_3_^2^	1	8.90	8.90	8.33	0.0073*	− 1.90
X_4_^2^	1	11.51	11.51	10.78	0.0027*	− 2.16
X_5_^2^	1	5.06	5.06	4.74	0.0378*	− 1.43
**Error effect**						
Lack of Fit	22	24.62	1.12	1.232454	0.4126	
Pure Error	7	6.36	0.91			

R^2^	0.9937	SD	1.03			
Adj R^2^	0.9894	Mean	84.29			
Pred R^2^	0.9834	C.V. %	1.23			
Adeq precision	50.65	PRESS	81.71			

*F* Fishers’s function, *P* level of significance, *C.V* coefficient of variation.

*Significant values.

**Table 4:** Fit summary for FCCCD for adsorption of Remazol brilliant blue dye by using *Gelidium corneum* biomass results.

Source	Sum of squares	*df*	Mean square	*F *value	*P *value *P*rob > *F*
**Lack of fit tests**					
Linear	3568.87	37	96.46	41.26	< 0.0001*
2FI	3088.76	27	114.40	48.93	< 0.0001*
Quadratic	31.07	22	1.41	0.60	0.8276
**Sequential model sum of squares**					
Linear vs Mean	1285.67	5	257.13	3.16	0.0161*
2FI vs Linear	480.11	10	48.01	0.53	0.8598
Quadratic vs 2FI	3057.69	5	611.54	373.84	< 0.0001*

Source	SD	R-squared	Adjusted R-squared	Predicted R-squared	PRESS
**Model summary statistics**					
Linear	9.03	0.2639	0.1803	0.0952	4407.12
2FI	9.56	0.3625	0.0813	− 0.2599	6136.87
Quadratic	1.28	0.9903	0.9835	0.9718	137.12

*df* degree of freedom, *PRESS* sum of squares of prediction error, *2FI* two factors interaction.

*Significant values.

**Table 5:** Fit summary for FCCCD for biosorption of Hg^2+^ by *Gelidium corneum* biomass results.

Source	Sum of squares	*df*	Mean square	*F *value	*P *value *P*rob > *F*
**Lack of fit tests**					
Linear	3595.54	37	97.18	107.03	< 0.0001*
2FI	3032.49	27	112.31	123.71	< 0.0001*
Quadratic	24.62	22	1.12	1.23	0.4126
**Sequential model sum of squares**					
Linear vs Mean	1330.25	5	266.05	3.25	0.0139*
2FI vs Linear	563.05	10	56.30	0.63	0.7778
Quadratic vs 2FI	3007.88	5	601.58	563.26	< 0.0001*

Source	SD	R-squared	Adjusted R-squared	Predicted R-squared	PRESS
**Model summary statistics**					
Linear	9.05	0.2697	0.1867	0.1019	4429.65
2FI	9.45	0.3839	0.1120	− 0.2033	5934.76
Quadratic	1.03	0.9937	0.9894	0.9834	81.71

*df* degree of freedom, *PRESS* sum of squares of prediction error, *2FI* two factors interaction.

*Significant values.

The values of the determination coefficient (R^2^) provide an indication of the degree of variation in the dependent variable (response values) that the experimental variables can explain. When a regression model's R-squared value is greater than 0.9 (closer to 1), it means the model is efficient and can accurately predict the response^36^. The regression model of Remazol brilliant blue dye has an R^2^ value of 0.9903. This means that 99.03 percent of the Remazol brilliant blue dye removal percentages variation were due to the process variables, and that the model could not explain only 0.97 percent of the overall variability (Table 2). Moreover, the Hg^2+^ model has an R^2^ value of 0.9937 (Table 3), which means that 99.37 percent of the Hg^2+^ removal percentage variations were due to the process variables and that the model could not explain only 0.63% of the overall variations. Moreover, Koocheki et al.^37^ conclude that a high R^2^ value does not actually imply that the regression model is a strong model, and this can only be assumed on the basis of a high value of the adjusted R^2^. The current Adj R^2^ values were found to be 0.9835 and 0.9894 for the removal of both Remazol brilliant blue dye and Hg^2+^; respectively (Tables 2, 3). A high value of adjusted R^2^ means that the model is highly significant and that the predicted and experimental findings of Remazol brilliant blue dye and Hg^2+^ removal using *Gelidium corneum* biomass as biosorbent are in strong agreement. In our study, the predicted R^2^ values of 0.9718 and 0.9834 for Remazol brilliant blue dye and Hg^2+^ removal percentage; respectively (Tables 2, 3). The high predicted R^2^ means high model significance in predicting the Remazol brilliant blue dye and Hg^2+^ removal percentage values. The adjusted and predicted R-squared values are in excellent agreement with each other. This revealed a strong agreement between the predicted and experimental findings of Remazol brilliant blue dye and Hg^2+^ removal percentage. Consequently, the model in the current study is appropriate for prediction of Remazol brilliant blue dye and Hg^2+^ removal % in the range of the process parameters that have been studied. *P* values were used as a tool for evaluating the statistical significance of each variable. The variables with *P* values less than 0.05 were assumed to have significant effects in this study^38^.

Analysis of variances (ANOVA) of the quadratic regression model of response Y_1_ [Remazol brilliant blue dye removal percentage] has a very low *P *value < 0.0001 with the Fisher's *F* test (*F *value = 147.43), which indicates that the regression model is very significant (Table 2). With probability values of < 0.05, it was apparent that the individual or linear effects of all processing parameters, including Hg^2+^ concentration (X_1_), Remazol brilliant blue dye concentration (X_2_), initial pH value (X_3_), contact time (X_4_) and *Gelidium corneum* biomass concentration (X_5_), were significant for removal of Remazol brilliant blue dye (Table 2). The mutual interactions between all the variables are significant with probability values of < 0.05 except the interactions between X_1_X_3_ (Hg^2+^ concentration and initial pH value), X_1_X_4_ (concentration of Hg^2+^ and contact time) and X_2_X_4_ (Remazol brilliant blue dye concentration and contact time) are not significant with *P *value > 0.05. In addition, coefficients' probability values indicate that the quadratic impact of Remazol brilliant blue dye concentration (X_2_) is not significant, whereas the quadratic effects of the other four variables are significant (Table 2).

Moreover, the ANOVA of the model of response Y_2_ [removal percentage of Hg^2+^] has a very low *P *value < 0.0001 with the Fisher's *F* test (*F *value = 229.45), which indicates that the regression model is very significant (Table 3). With probability values of < 0.05, it was apparent that the individual or linear effects of all process parameters were significant for removal of Hg^2+^ (Tables 3). The mutual interactions between all the variables are significant with probability values of < 0.05 except the interactions between X_1_X_3_ (Hg^2+^ concentration and initial pH level), X_2_X_4_ (Remazol brilliant blue dye concentration and contact time) and X_3_X_4_ (initial pH value and contact time) with *P *value > 0.05 are not significant. Furthermore, the quadratic effects are significant for all five variables (Table 2).

The coefficient of variance percent for Remazol brilliant blue dye removal (percent) findings is comparatively low (C.V. = 1.81%), implying that the performed trials were of high precision and consistency^39^. The level of noise is defined by adequate precision; a level greater than 4 is desired and demonstrates the model's durability. The new Remazol brilliant blue dye removal model had an acceptable precision ratio of 47.30, indicating that the model is valid. The Remazol brilliant blue dye removal (percent) model has an estimated residual error number of squares (PRESS) value of 137.12. The average and standard deviation of the model are 70.70 and 1.28, respectively.

The coefficient of variance percentage for Remazol brilliant blue dye removal (percent) findings is comparatively low (C.V. = 1.81 percent), implying that the trials were carried out with high precision and accuracy^39^. Adequate precision defines the level of noise; a level greater than 4 is desirable and indicates the model's reliability. The precision ratio of the Remazol brilliant blue dye removal model is 47.30, indicating that the model is valid. Remazol brilliant blue dye removal (percent) model analysis has a PRESS (predicted residual error sum of squares) value of 137.12. The standard deviation model's and mean values are respectively 1.28 and 70.70 (Table 2). At the same time, the coefficient of variance percentage for Hg^2+^ removal (percent) finding is very low (C.V. = 1.23 per cent), which indicates the high precision and accuracy of the conducted experiments. The present Hg^2+^ model had an adequate precision ratio of 50.65, indicating that the model is appropriate. The PRESS value for the Hg^2+^ removal results analysis is 81.71. The standard deviation model's and mean values are 1.03 and 84.29; respectively (Table 3).

The results of the fit summary (Tables 4, 5) were used to select the optimal highly significant polynomial model from linear, 2FI (the interaction of each pair-wise combination of the variables) and quadratic models fitting the FCCCD experimental data. The appropriate model, which would give the experimental results close to the predicted values by the model with the lowest residual values, was selected based on an insignificant lack of fitness test and significant model parameters^36^. The appropriate model for Hg^2+^ and Remazol brilliant blue dye removal was the quadratic model (Tables 4, 5). The results of the fit summary demonstrated that the quadratic models used for simultaneous bioremoval of both Hg^2+^ and Remazol brilliant blue dye by the dry *Gelidium corneum* biomass are highly significant models having significant model terms with a very low probability value (*P *value < 0.0001). Furthermore, lack of fit test revealed that the quadratic models of both simultaneous bioremoval of both Hg^2+^ and Remazol brilliant blue dye by the dry *Gelidium corneum* biomass are non-significant with high probability value of 0.8276, 0.4126 and *F* value of 0.60, 1.23 for Hg^2+^ and Remazol brilliant blue dye removal percentages; respectively. For Remazol brilliant blue dye removal, the quadratic model's summary statistics revealed the highest adj. (0.9835) and pred. (0.9718) R^2^ and the lowest standard deviation of 1.28. In addition, the quadratic model's summary statistics for Hg^2+^ removal had the highest adj. (0.9894) and pred. (0.9834) R^2^ and the lowest standard deviation of 1.03.

The optimum levels of the chosen independent factors (Hg^2+^ concentration (X_1_), Remazol brilliant blue dye concentration (X_2_), initial pH level (X_3_), contact time (X_4_) and *Gelidium corneum* biomass concentration (X_5_) giving the maximum simultaneous bioremoval of Hg^2+^ and Remazol brilliant blue dye were evaluated by a second-order polynomial equation. The regression coefficients can be used to predict the removal percentages of Remazol brilliant blue dye and Hg^2+^ using the following second-order polynomial regression equations:

1 $$\begin{aligned} & {\text{The}}\;{\text{predicted}}\;{\text{value}}\;{\text{of}}\;{\text{Remazol}}\;{\text{brilliant}}\;{\text{blue}}\;{\text{dye}}\;{\text{removal}}\left( \% \right) \\ & \quad = + 82.89 + 0.78{\text{X}}_{1} + 5.73{\text{X}}_{2} {-}0.65{\text{X}}_{3} + 0.78{\text{X}}_{4} + 1.83{\text{X}}_{5} \\ & \quad \quad + 0.76{\text{X}}_{1} {\text{X}}_{2} + 0.04{\text{X}}_{1} {\text{X}}_{3} + 0.24{\text{ X}}_{1} {\text{X}}_{4} + 0.78{\text{ X}}_{1} {\text{X}}_{5} + 0.58{\text{X}}_{2} {\text{X}}_{3} \\ & \quad \quad + 0.06{\text{ X}}_{2} {\text{X}}_{4} - 1.64{\text{X}}_{2} {\text{X}}_{5} - 1.32{\text{ X}}_{3} {\text{X}}_{4} - 2.46{\text{ X}}_{3} {\text{X}}_{5} - 1.70{\text{ X}}_{4} {\text{X}}_{5} \\ & \quad \quad - 6.27{\text{X}}_{1}^{2} + 0.34{\text{X}}_{2}^{2} - 7.05{\text{X}}_{3}^{2} - 3.17{\text{X}}_{4}^{2} - 1.77{\text{X}}_{5}^{2} \\ \end{aligned}$$

2 $$\begin{aligned} & {\text{The}}\;{\text{predicted}}\;{\text{value}}\;{\text{of}}\;{\text{Hg}}^{2 + } \;{\text{removal}}\left( \% \right) \\ & \quad = + 96.44 + 1.68{\text{X}}_{1} + 5.70{\text{X}}_{2} {-}1.34{\text{X}}_{3} + 1.24{\text{X}}_{4} \\ & \quad \quad + 0.71{\text{X}}_{5} + 0.98{\text{X}}_{1} {\text{X}}_{2} + 0.30{\text{X}}_{1} {\text{X}}_{3} - 1.03{\text{X}}_{1} {\text{X}}_{4} \\ & \quad \quad - 1.25{\text{X}}_{1} {\text{X}}_{5} - 1.0{\text{X}}_{2} {\text{X}}_{3} - 0.32{\text{X}}_{2} {\text{X}}_{4} - 2.26{\text{X}}_{2} {\text{X}}_{5} \\ & \quad \quad + 0.35{\text{X}}_{3} {\text{X}}_{4} - 2.24{\text{X}}_{3} {\text{X}}_{5} - 1.61{\text{X}}_{4} {\text{X}}_{5} - 6.24{\text{X}}_{1}^{2} \\ & \quad \quad - 6.14{\text{X}}_{2}^{2} + 1.9{\text{X}}_{3}^{2} - 2.16{\text{X}}_{4}^{2} - 1.43{\text{X}}_{5}^{2} \\ \end{aligned}$$

where X_1_–X_5_ are the coded levels of Remazol brilliant blue dye conc., Hg^2+^ conc., *Gelidium corneum* biomass conc., initial pH value, and contact time; respectively.

The abovementioned second-order quadratic equations (1, 2) demonstrate how the independent factors, their quadratic as well as their interactions between them have influenced Remazol brilliant blue dye and Hg^2+^ removal percentages from aqueous solution by dry biomass of the alga *Gelidium corneum* as biosorbent. The negative coefficient values indicate that the quadratic, linear or interaction effects amongst the factors possess negative impacts on Remazol brilliant blue dye and Hg^2+^ removal percentages (i.e., reduces the removal %), while the positive coefficient values mean that the linear, quadratic or interaction effects amongst various variables maximize the removal percentages of Remazol brilliant blue dye and Hg^2+^ from the aqueous solutions in the tested levels of the process variables.

### Effects of each pair of process variables on Remazol brilliant blue dye and Hg^2+^ removal percentages (three-dimensional surface plots)

The 3D (three-dimensional surface) plots were created to illustrate the correlation between the responses (Y_1_: Remazol brilliant blue dye removal percentages, Y_2_: Hg^2+^ removal percentages) and the five the surveyed independent factors, to evaluate changes in the responses and to find the best levels of the independent variables for maximum Hg^2+^ and Remazol brilliant blue dye removal from binary solution. The three-dimensional plots for the pair-wise combination of the five independent variables (Hg^2+^ concentration, Remazol brilliant blue dye concentration, contact time, initial pH value and algal biomass concentration) have been created by plotting the Remazol brilliant blue dye removal (percent) or Hg^2+^ removal (percent) on Z-axis against two independent variables while keeping all other variables at their zero levels. As a result, ten 3D plots for each response [Y_1_: Remazol brilliant blue dye removal (%), Y_2_: Hg^2+^ removal were created.

**Figure 2:**
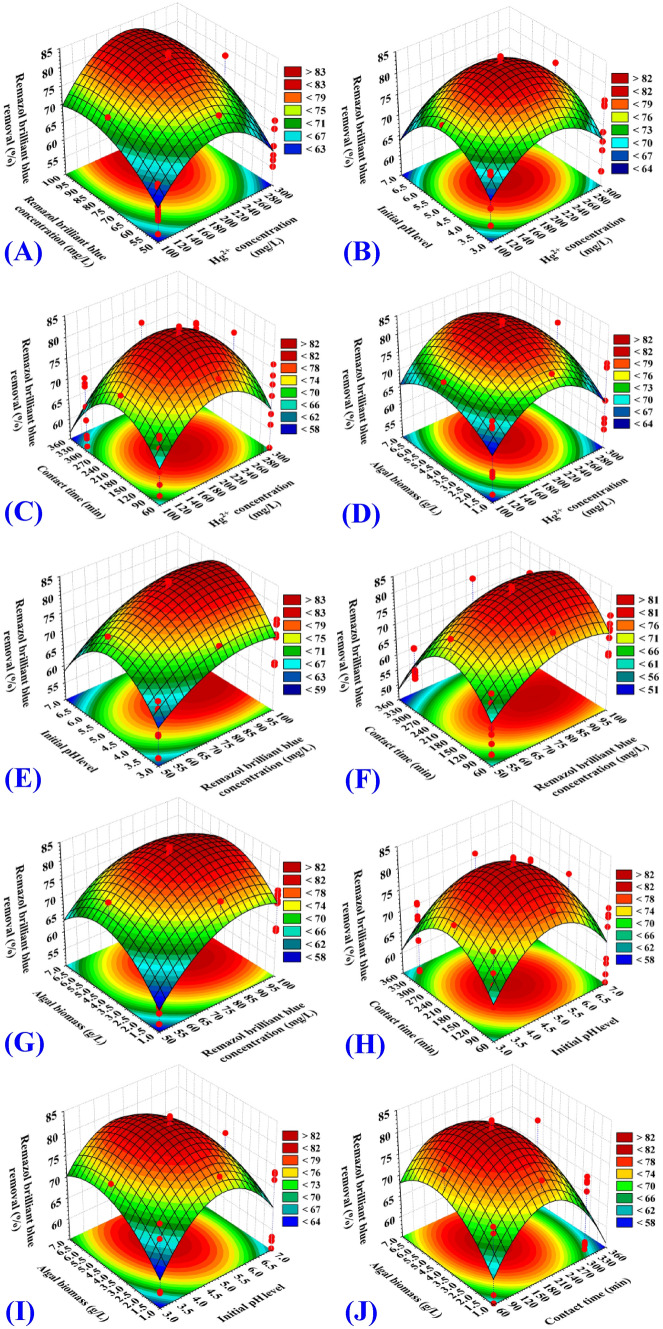
Three-dimensional surface plot for biosorption of Remazol brilliant blue dye by *Gelidium corneum* biomass, showing the interactive effects of the five tested variables.

**Figure 3:**
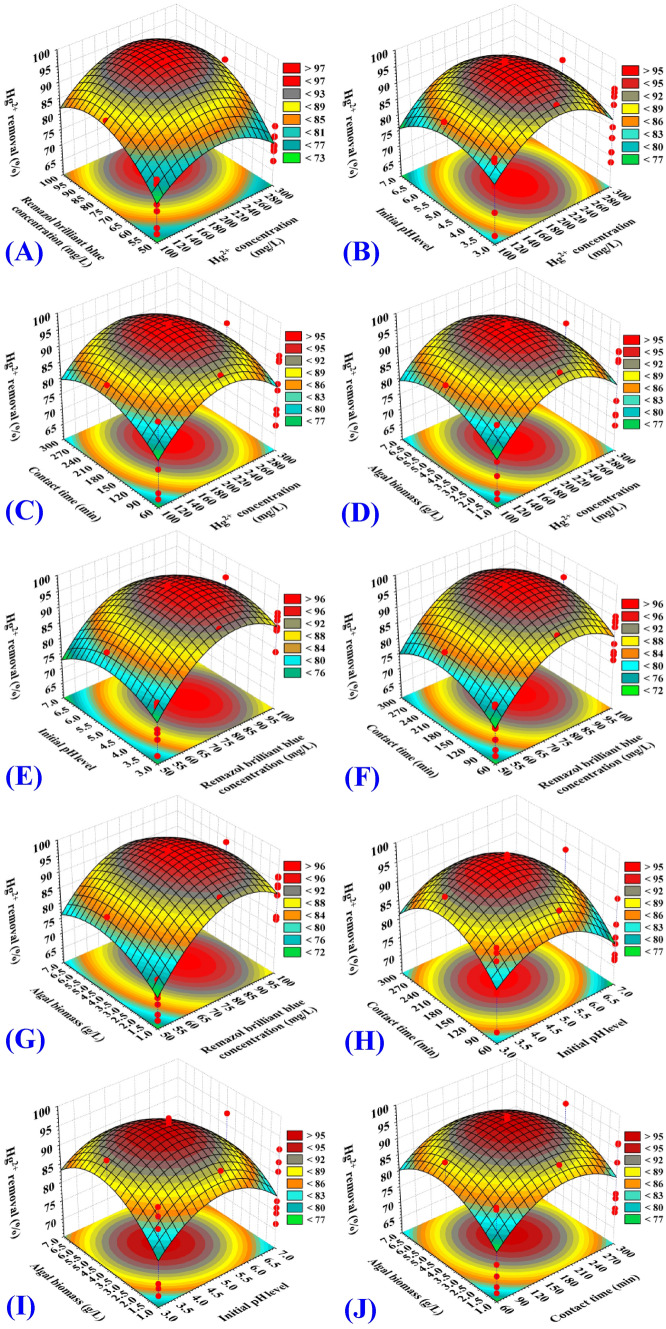
Three-dimensional surface plot for biosorption of Hg^2+^ by *Gelidium corneum* biomass, showing the interactive effects of the five tested variables.

The 3D plots (Figs. 2A, 3A) demonstrate the mutual effects of initial Hg^2+^ (X_1_) and Remazol brilliant blue dye concentrations (X_2_) that occur simultaneously on Remazol brilliant blue dye and Hg^2+^ removal efficiencies (%), while initial pH value, incubation time and *Gelidium corneum* biomass concentration (X_3_–X_5_) were kept at their zero levels. Figure 2A demonstrates that as the initial concentrations of Remazol brilliant blue dye and Hg^2+^ increased, the percentage of dye removed by *Gelidium corneum* biomass increased, implying that the biosorption process was strongly dependent on the initial levels of both Remazol brilliant blue dye and Hg^2+^. Figure 3A demonstrates that the percentage of Hg^2+^ removed increased as the concentration of Hg^2+^ increased, and that the initial Hg^2+^ concentration had a significant effect on the percentage of Hg^2+^ removed by *Gelidium corneum* biomass, and that this effect was positive.

However, as the Hg^2+^ concentration rises, the removal percentage decreases, indicating that the biosorption process was strongly dependent on the Hg^2+^ concentration at the start. This may be due to the presence of free unsaturated binding sites on the algae biomass surface at the start, which resulted in high Hg^2+^ biosorption. As well, the initial Hg^2+^concentration increased the driving force, which was needed to overcome the increase in the mass transfer resistance that existed between the surface of the alga and the aqueous binary solution^40^. Thereafter, a further increase in the Hg^2+^ concentration, the Hg^2+^ removal percentage decreased. This is due to the full occupation of the adsorption sites on the algal biomass surface.

### Effect of initial Hg^2+^ concentration on Hg^2+^ biosorption by *Gelidium corneum* biomass

With increasing Hg^2+^ concentrations, the biosorption process of Hg^2+^ by *Gelidium corneum* biomass increased. At an initial Hg^2+^ concentration of 200 mg/L, a maximum Hg^2+^ removal with percentage of 98.25 percent was achieved. A rise in the amount of biosorbed Hg^2+^ as a result of an increase in the initial Hg^2+^ level may be attributed to an increase in the driving force of Hg^2+^ needed to overcome the existing mass transfer resistance between *Gelidium corneum* biomass surface and the aqueous solution^40^. The decrease in the percentage of Hg^2+^ removal at greater concentrations can also be explained by the saturation of binding sites available on the *Gelidium corneum* biomass, the number of accessible functional groups on the algal biomass was limited, and the amount of Hg^2+^ ions increased.

The 3D plots (Figs. 2B, 3B) demonstrate the mutual effects of initial Hg^2+^ concentration (X_1_) and initial pH level (X_3_) on Remazol brilliant blue dye and Hg^2+^ removal efficiencies (%), while initial Remazol brilliant blue dye concentration, contact time and algal biomass concentration (X_2_, X_4_, X_5_) have been maintained at zero levels. The 3D plots (Figs. 2B, 3B) demonstrate that the removal efficiencies of both Remazol brilliant blue dye and Hg^2+^ by *Gelidium corneum* biomass increased linearly with increasing Hg^2+^ concentration, achieving the maximum efficiency at its center level. Furthermore, as the concentration of Hg^2+^ continues to increase, the removal percentages of both Remazol brilliant blue dye and Hg^2+^ gradually decrease.

The removal percentages of both Remazol brilliant blue dye and Hg^2+^ were improved when the pH of the solution was raised up to pH 5. The removal percentages decreased as the initial pH was raised higher. The interaction effect between X_1_X_3_ (concentration of Hg^2+^ and initial pH value) is not significant (*P *value > 0.05) for Remazol brilliant blue dye and Hg^2+^ removal percentages (Tables 2, 3).

### Effect of initial Remazol brilliant blue dye concentration on the biosorption process

With increasing concentrations of Remazol brilliant blue dye, the biosorption process of Remazol brilliant blue dye by *Gelidium corneum* biomass increased. The maximum removal percent of Remazol brilliant blue dye was 89.18 percent at an initial Remazol brilliant blue level of 99.33 mg/L. The dye's percentage removal and biosorption efficiency are strongly reliant on the dye's initial concentration^41^. The effect of the initial concentration of the dye is based on the number of binding active sites accessible on the adsorbent's surface^42^. When the initial dye concentration is increased, the percentage of dye removed generally decreases, which could be attributed to adsorption sites on the adsorbent surface being saturated^43^.

### Effect of initial pH level on Hg^2+^ biosorption process by *Gelidium corneum* biomass

The pH level was established as the key process factor that affects the process of biosorption. The solution pH has a well-recognized effect on the solubility of the metal ions and affects the degree of the metals and biomass ionization and the functional groups activities through the processes of biosorption^44^. The red algae's cell walls are made up of mannan fibrils, xylan, cellulose, and substantial polysaccharide matrix, including mucusy sugars (gelans), agar, carrageenan, and proteins, alongside minerals.

Certain red algae can take up seawater calcium and accumulate calcium carbonate in their bodies^34^. Such components provide a variety of functional groups that can serve as binding sites for contaminant ions. The biomass has been verified as natural ion exchange materials containing functional groups (either acidic or basic). The net charge on marine algae as biosorbent cell surfaces is pH dependent since their cell wall surfaces are composed of polysaccharides^45^.

In this study, mercury is a cationic metal, present in a positively charged state, Hg^2+^ (mercuric). The biosorption of Hg^2+^ by *Gelidium corneum* biomass was low at lower acidic pH values (pH < 5), which can be explained by higher positively charged H^+^ ions (protons) on the surface of a biosorbent competing with cations (metal ions). As pH increase, Hg^2+^ biosorption of by *Gelidium corneum* biomass increased, and the optimum pH value for maximal removal of Hg^2+^ was about pH 5.

Lower acidic pH values cause protonation (positive charge) of binding sites in the biomass, resulting in repulsion between the binding sites on the biosorbent cell surface and cations of heavy metal. As a result, competition for binding sites among high concentrations of H^+^ ions (protons) and cations of heavy metals reduces biosorption of heavy metals^44^. On the other hand, the binding sites are deprotonated at high pH values and result in the formation of various negative charged functional groups including carboxyl, amine, and hydroxyl on the biosorbent surface. Consequently, the functional groups with a negative charge encourage and increase the biosorption of positively charged heavy metal ions^46^. An ion exchange process appears to be involved in the biosorption of Hg^2+^. The cationic positively charged Hg^2+^ binds to the binding sites on the negatively charged *Gelidium corneum* biomass surface by replacing two acidic OH^−^ ions which cause improved biosorption of Hg^2+^ at higher pH values. The decrease in the Hg^2+^ removal percentage at higher pH values may be caused by the formation of increased quantities of OH^−^ ions in the solution, which cause the reaction of the metal ions with OH^−^ ions and the precipitation of the metal hydroxide.

### Effect of initial pH value on dye biosorption process by *Gelidium corneum* biomass

The biosorption process of dye molecules is highly pH-dependent as the pH of aqueous dye solution plays a crucial effect on the surface binding sites of the biosorbent as well as the degree of dye molecule ionization process^47,48^. Therefore, it is necessary to set up the pH of the solution in order to obtain proper surface charge functional groups that interact with the dye^49^. Remazol brilliant blue dye is negatively charged.

At lower acidic pH values, the surfaces of the biosorbent are protonated and gain net positive charges, so that the binding of negatively charged (anionic) dyes to the biosorbent surfaces increases^50,51^. Maximum removal of Remazol brilliant blue dye at a low pH value could be due to the electrostatic interactions between the molecules of negatively charged Remazol brilliant blue dye anions and H^+^ ions from the positively charged alginate surface. The biosorbent must be attributable to the interaction of sulfonic (–SO^3−^) groups of Remazol brilliant blue dye with –OH groups on the biosorbent surface^52,53^.

The 3D plots (Figs. 2C, 3C) show the simultaneous impact of initial Hg^2+^ concentration (X_1_) and the contact time (X_4_) on Remazol brilliant blue dye removal (%) and Hg^2+^ removal (%), while initial Remazol brilliant blue dye concentration, initial pH value and algal biomass concentration (X_2_, X_3_, X_5_) were held at central levels. The percentage removal of Remazol brilliant blue dye (Fig. 2C) and Hg^2+^ (Fig. 3C) by *Gelidium corneum* biomass increased with increasing Hg^2+^ concentration and contact time, reaching a maximum at their center levels. However, further increasing in Hg^2+^ concentration or the contact time leads to a gradual decrease in both Remazol brilliant blue dye and Hg^2+^ removal percentages.

### Effect of contact time

Rapid sorption during the initial contact period may be due to the presence of a large number of vacant active sites^54^. Experimental findings have clearly shown that with the increase of contact time to the optimum, the removal percentages of Hg^2+^ and Remazol brilliant blue dye by *Gelidium corneum* biomass increases. The maximum biosorption percentages can be attributed to the accessibility of a suitable surface area of *Gelidium corneum* biomass for biosorption and the existence of vacant active sites and also the high concentrations of Hg^2+^ and Remazol of brilliant blue. However, as time increases, the active binding sites on the biomass surface have been saturated, causing the adsorption slower^55^.

### Effect of initial concentration of the algal biomass

The number of unsaturated active sites available for the biosorption process increased as the algal biomass concentration was increased, resulting in an increase in Remazol brilliant blue dye and Hg^2+^ removal percent. According to Phugare et al.^56^, the increase in removal percentage is anticipated due to a larger biosorbent surface area, which increases the number of active sites, resulting in effective biosorption. The percentage removal of Remazol brilliant blue dye and Hg^2+^ decreased as the algal biomass concentration was increased from 3.08 to 7 g/L. According to Garg et al.^57^, the decrease in removal percentage after the optimal dose was attained was due to the saturation of all binding sites. Biomass agglomeration could be a cause of decreased removal efficacy at elevated algal biomass concentrations, which could reduce the total available effective surface area needed for the biosorption process. El-Naggar et al.^19^ reported that agglomeration can reduce the effective biomass surface area, which can reduce the efficiency of the biosorption process at high concentrations of algal biomass. On the other hand, the agglomeration limits the active sites for dye and metal ions removal^58^. However, EL Hassouni et al.^59^ indicated that high algal biomass concentrations, leading to lower removal percentage due to the increasing number of active sites uncovered due to inadequate metal ions available for linkage with all the active sites.

The main reasons for choosing biomass for biosorption process uses are availability and low cost. Algae have been shown to be efficient, sustainable, low-cost, promising forms of biosorbent for the removal of various dyes and metals due to their surface properties, local abundance in marine and freshwater environments, high capacity for metals removal, regeneration, and reuse of biomass^21,23,60,61^. On the other hand, Davis et al.^62^ demonstrated that algae have higher metal binding ability due to the existence of cell wall lipids, polysaccharides or proteins with functional groups such as amino, carboxylic and hydroxyl, which may serve as metal binding sites. There are some reports on the biosorption of Remazol brilliant blue dye by white rot fungi, *Polyporus* sp. S133^12^, onto immobilized green microalga, *Scenedesmus quadricauda*^63^, biochar derived from the green seaweed, *Ulva lactuca* biomass^64^ and biochar derived from biomass of brown seaweed *Turbinaria conoides*^65^ with varying levels of removal efficiency. The biosorption potential of each alga for Remazol brilliant blue dye differed. This can be explained by the variation in polysaccharide and protein composition in cell walls that provide binding sites for the cell surface.

The 3D plots (Figs. 2D, 3D) show the combined impacts of initial Hg^2+^ concentration (X_1_) and algal biomass concentration (X_5_) on Remazol brilliant blue dye removal (%) and Hg^2+^ removal (%), while the initial level of Remazol brilliant blue dye, initial pH value and incubation time (X_2_, X_3_, X_4_) have been held at their central levels. Remazol brilliant blue dye (Fig. 2D) and Hg^2+^ (Fig. 3D) removal percentages were increased by increasing the initial Hg^2+^ concentration and *Gelidium corneum* biomass concentration. Increases in Hg^2+^ concentration above 216.87 mg/L and algal biomass concentrations above 3.08 g/L decreased both Remazol brilliant blue dye and Hg^2+^ removal percentages.

The 3D plots (Figs. 2E, 3E) indicate Hg^2+^ and Remazol brilliant blue dye removal (%) as a result of variations in Remazol brilliant blue dye (X_2_) and initial pH (X_3_) levels while other factors have been held at their central levels. The removal percentages of Remazol brilliant blue dye (Fig. 2E) and Hg^2+^ (Fig. 3E) increase with an increasing initial pH value. Also, increasing the Remazol brilliant blue dye concentration (X_2_) resulted in increased Remazol brilliant blue dye and Hg^2+^ removal. The removal percentage of both Remazol brilliant blue dye and Hg^2+^ decreased as the initial Remazol brilliant blue dye concentration increased above 99.33 mg/L and the initial pH value increased above 4.85.

Figures 2F and 3F show that, maximum removal percentage of both Remazol brilliant blue dye and Hg^2+^ were obtained by using 99.33 mg/L of initial Remazol brilliant blue dye concentration (X_2_) and 210.34 min of contact time (X_4_). Similarly, Figs. 2G and 3G reveal that increased initial Remazol brilliant blue dye concentration (X_2_) and algal biomass concentration (X_5_) resulted in higher removal percentages for Remazol brilliant blue dye and Hg^2+^. The maximum percentage removal for both Remazol brilliant blue dye and Hg^2+^ were obtained using an initial pH level of 4.77, as shown in Figs. 2H and 3H. The interaction impacts of initial pH level and algal biomass concentration are obvious in Figs. 2I, 3I. Figures 2J and 3J reveal the effects of both contact time and algal biomass concentration.

### The model adequacy for Remazol brilliant blue dye removal (%) data analysis

**Figure 4:**
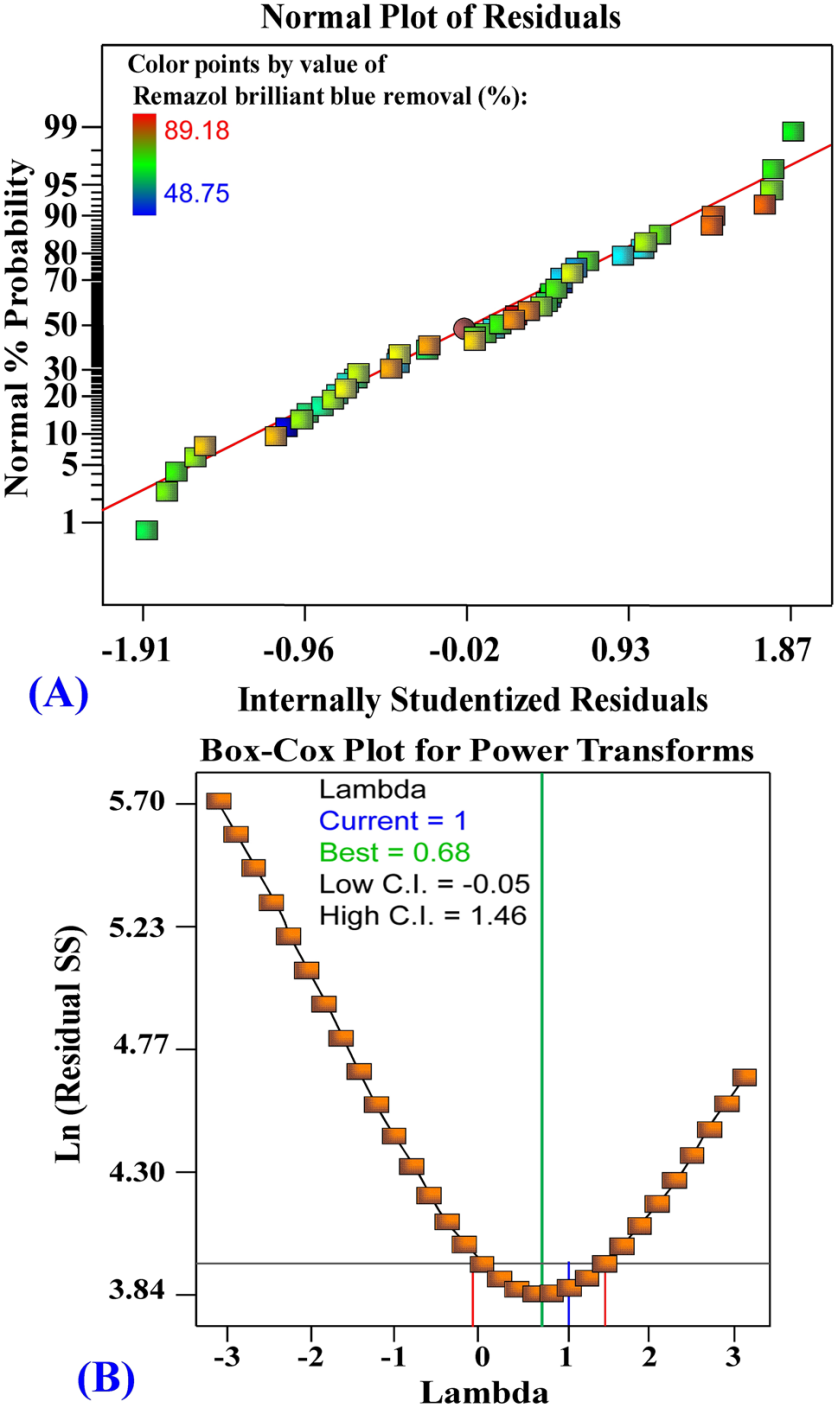
(**A**) Normal probability plot of internally studentized residuals, and (**B**) Box–Cox plot of model transformation of biosorption of Remazol brilliant blue dye by *Gelidium corneum* biomass.

The normal probability plot (NPP) is a critical aspect in checking the model's adequacy^66,67^. NPP of the residuals for Remazol brilliant blue dye removal (%) data analysis (Fig. 4A). The residuals of the model fitted are typically distributed along the standard distribution's diagonal line. This proves that the model is appropriate. As can be seen in the Box-Cox plot, the optimal value of Lambda (λ) value of 1 is between the two vertical red lines, which implies no transformation of data is necessary (Fig. 4B).

### The model adequacy for Hg^2+^ removal (%) data analysis

**Figure 5:**
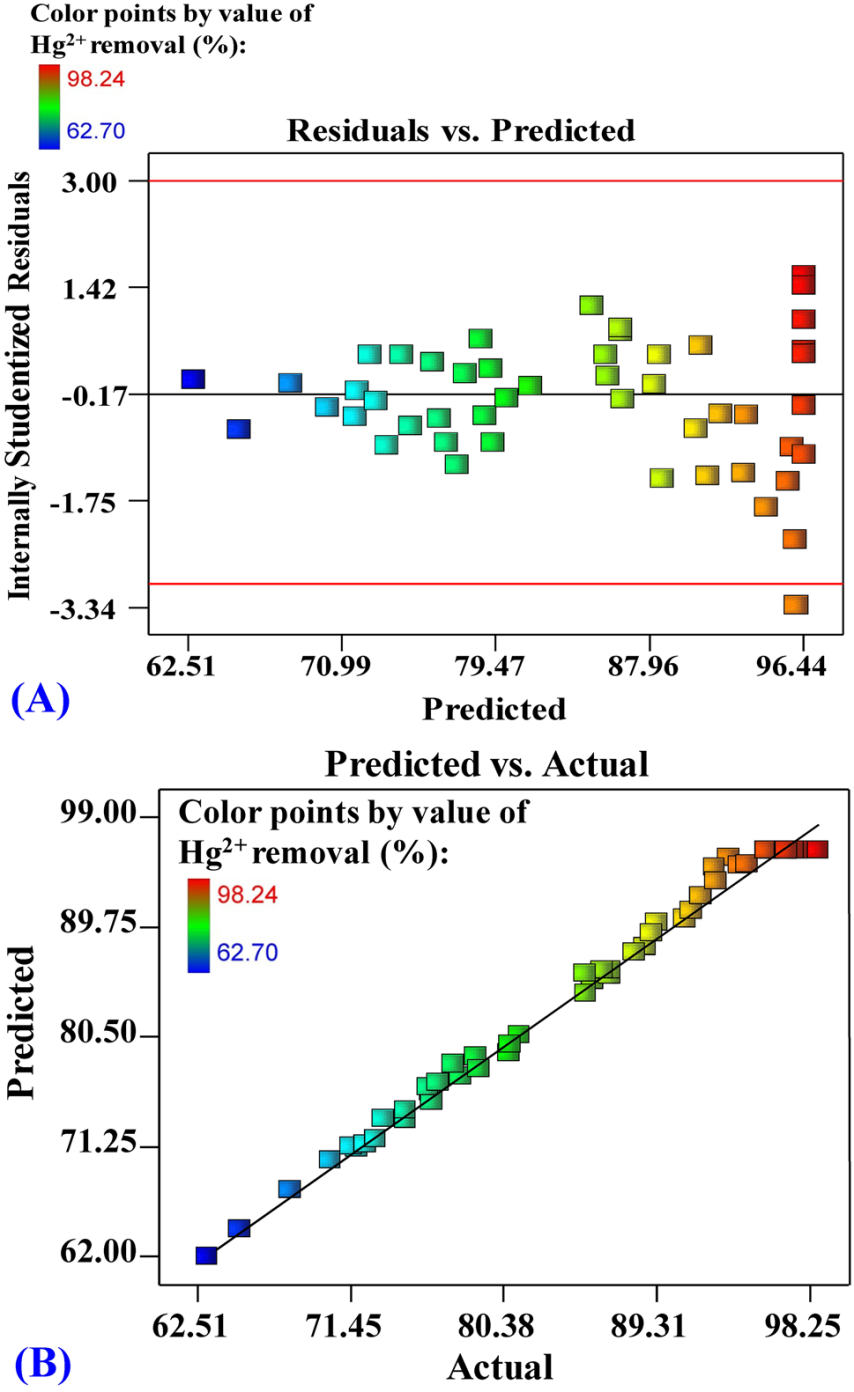
(**A**) Plot of internally studentized residuals versus predicted values, and (**B**) plot of predicted versus actual values of biosorption of Hg^2+^ by *Gelidium corneum* biomass.

The residuals versus expected values of the Hg^2+^ removal (percent) data analysis is plotted in Fig. 5A. The gathered points along the diagonal line indicate the model's validity, as seen in the graph. The actual versus predicted removal percentages of Hg^2+^ from binary solution by *Gelidium corneum* biomass are shown in Fig. 5B. Figure 5B depicts all of the predicted removal percentage points along the diagonal axis, demonstrating that the predicted Hg^2+^ removal percentages match the actual removal percentages, implying that the model is valid.

### Desirability function (DF)

**Figure 6:**
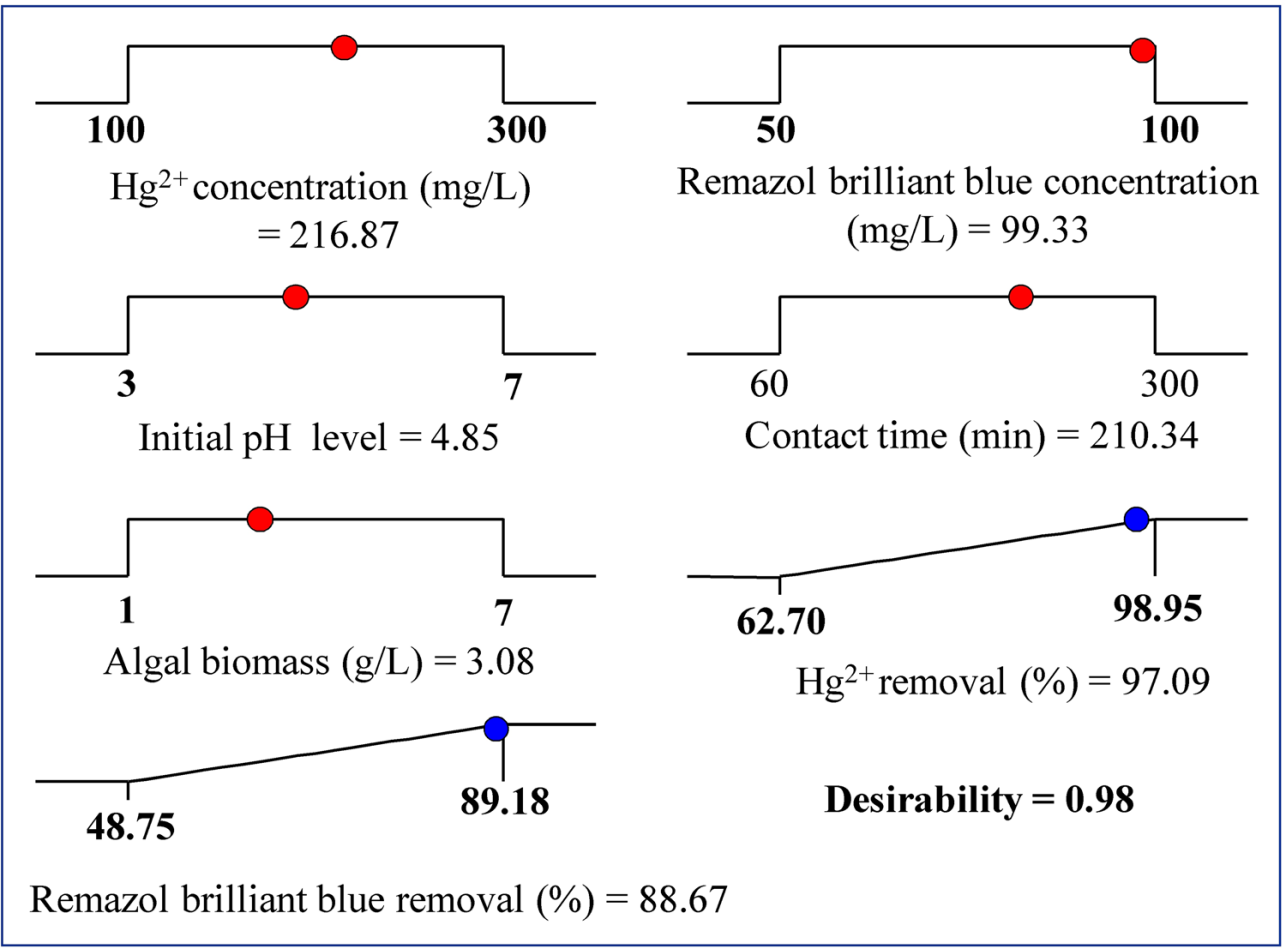
The optimization plot displays the desirability function and the optimum predicted values for the maximum simultaneous biosorption of Hg^2+^ and Remazol brilliant blue dye by *Gelidium corneum* biomass.

The tool of the desirability function (DF) in the Software Design Expert (version 7) was used for the optimization process. The desirability function (DF) was used to determine the best predicted conditions for maximum response. Its values ranged from zero to one^68^. The optimum predictable conditions for the maximum removal of Remazol brilliant blue dye and Hg^2+^ by *Gelidium corneum* biomass were 99.33 mg/L initial Remazol brilliant blue dye concentration, 216.87 mg/L initial Hg^2+^ concentration, initial pH level of 4.85, contact time of 210.34 min, and *Gelidium corneum* biomass concentration of 3.08 g/L (Fig. 6). Under these conditions, Hg^2+^ was removed at a rate of 97.09 percent and Remazol brilliant blue dye was removed at a rate of 88.67 percent with a DF of 0.98. The removal percentages of Remazol brilliant blue dye and Hg^2+^ by *Gelidium corneum* biomass were checked in triplicate under the optimum predicted conditions of the process variables, and the results of the experiments were compared to the expected values. In the experimental results, the average removal percentages of Remazol brilliant blue dye and Hg^2+^ were 90 percent and 98.65 percent; respectively. The verification revealed that the observed and predicted values were very close. The verification signifies that the DF accurately predicts the optimum conditions.

### Gelidium corneum biomass characterization

SEM, FTIR spectra, and EDS analyses were used to investigate the morphological features and surface properties of *Gelidium corneum* biomass before and after biosorption of Remazol brilliant blue dye and Hg^2+^.

### Scanning electron microscopy (SEM) for surface morphology examination

**Figure 7:**
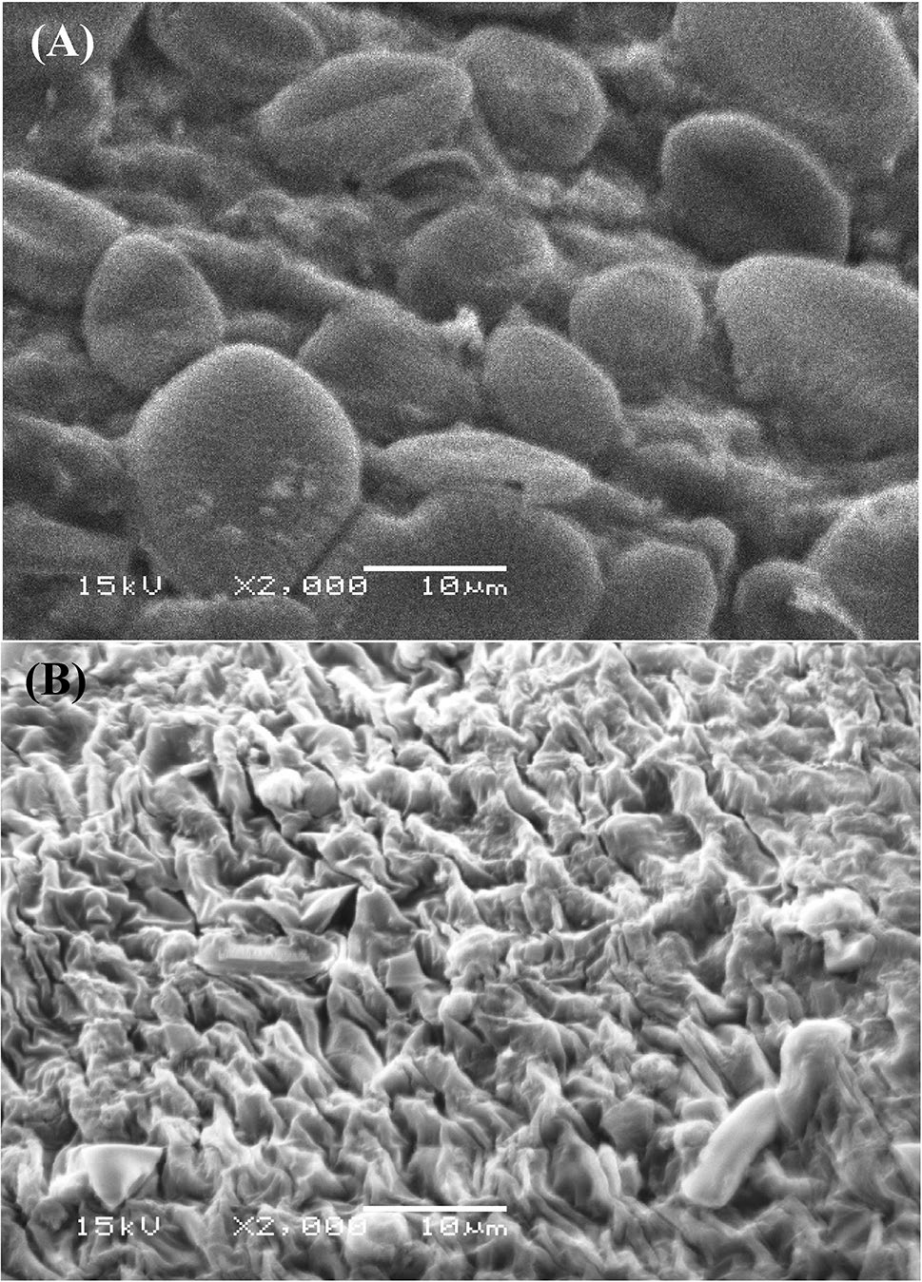
SEM micrograph of *Gelidium corneum* biomass: (**A**) before and (**B**) after simultaneous biosorption of Hg^2+^ and Remazol brilliant blue dye.

Before the biosorption process of Remazol brilliant blue dye and Hg^2+^, a micrograph of *Gelidium corneum* biomass surface (Fig. 7A) revealed a regular surface. Figure 7B shows the presence of new shiny large adsorbate ion particles on the surface of *Gelidium corneum* biomass after the biosorption process of Remazol brilliant blue dye and Hg^2+^), which were not present on the algal surface prior to the biosorption process. The cell surfaces showed obvious morphological changes, such as surface shrinkage. Strong cross-linking binding between negatively charged active sites in the polymers that make up their cell walls and Hg^2+^ may be the cause of these changes^69^.

Figure 7A showed the presence of Diatom shells on the surface of the algae. Silicon (Si) was detected by SEM–EDS analysis of *Gelidium corneum* biomass. Diatom shells were observed on the surface of the algae in a systematic manner. There was a lot of variation in the number of diatom shells found; some areas had a lot, while others had no diatoms^70^. The cell wall of diatoms is made up of silica, which is mixed with polysaccharide and protein. Due to the fact that silicate groups are the main component of these microorganisms, high amounts of silicon were observed in the areas where the outer diatom shells were found^70^.

### FTIR spectra analysis

**Figure 8:**
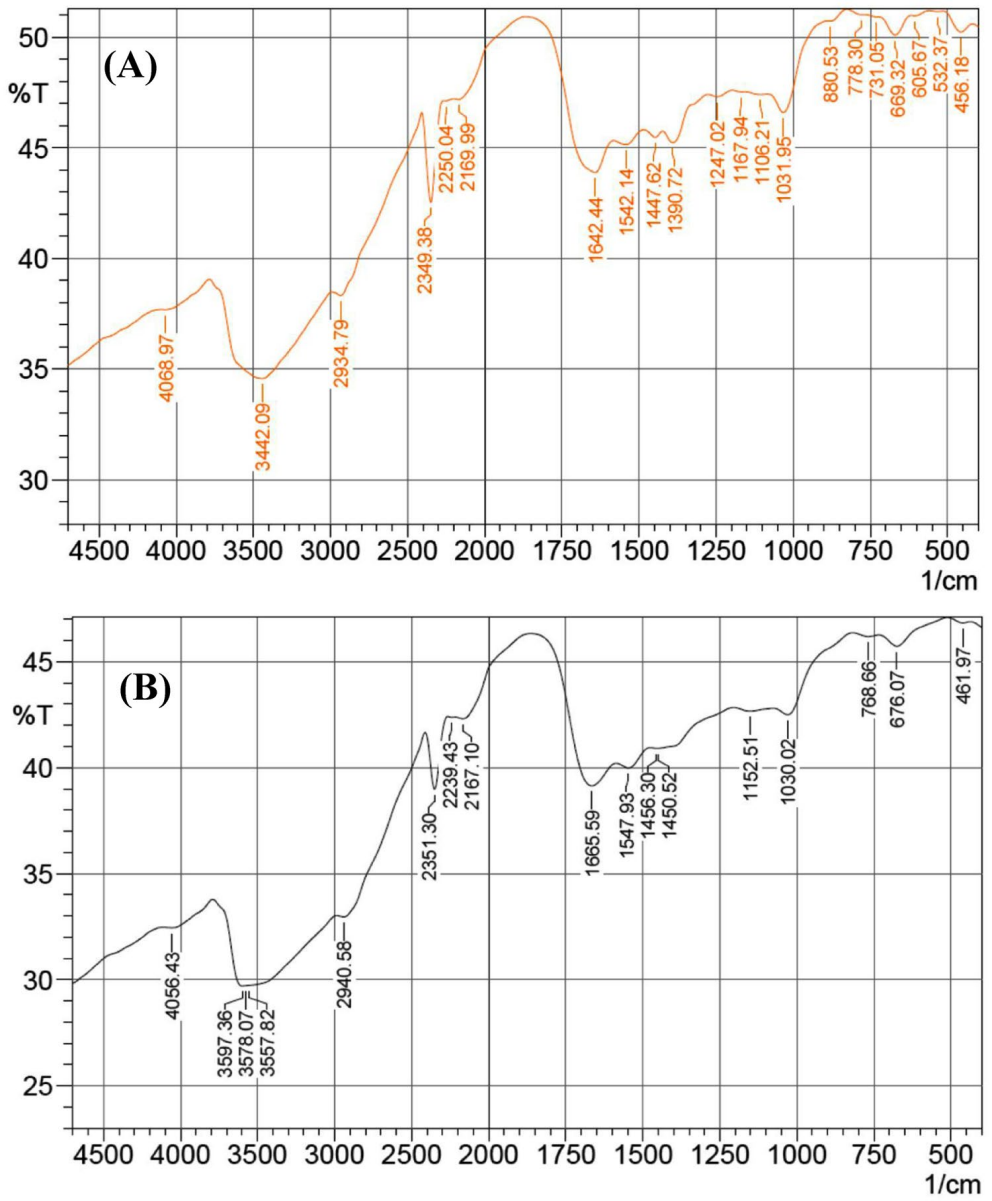
FTIR analysis of *Gelidium corneum* biomass: (**A**) before and (**B**) after simultaneous biosorption of Hg^2+^ and Remazol brilliant blue dye.

Fourier transform infrared (FTIR) spectroscopy analysis of the *Gelidium corneum* biomass was performed before and after the biosorption process of both Remazol brilliant blue dye and Hg^2+^. The FTIR spectra of both samples are shown in Fig. 8 and Table 6. The spectral data categorized the characteristic groups of *Gelidium corneum* biomass before and after the biosorption process of Remazol brilliant blue dye and Hg^2+^. The spectral data reveals any differences in morphological features and surface properties caused by interactions between the functional groups with Remazol brilliant blue dye and mercury ions. Generally, FTIR was applied to investigate the characteristic groups of the biomass after removal of the heavy metal ions^23^. The values of the bands may be shifted due to the interaction of the *Gelidium corneum* surface with the adsorbed ions of mercury that results in a shift, absence or change in the values of the characteristic bands in the FTIR spectrum.

**Table 6:** FTIR spectral analysis of *Gelidium corneum* biomass before and after biosorption of Hg^2+^ and Remazol brilliant blue dye.

Before biosorption		After biosorption		Shift	References
Wave no. (cm^−1^)	Annotations	Wave no. (cm^−1^)	Annotations		
4069	–OH hydroxyl groups stretching	4056	–OH hydroxyl groups stretching “alcohol”	13	Bhattacharya et al.^71^
3442	–OH or N–H stretching	3597, 3578, 3557	Strong broad –OH stretching	− 155	Li et al.^72^
2934	C–H stretching of aliphatic hydrogens or OH stretching	2940	Medium C–H stretching “alkane”	− 6	Zhao et al.^73^
2349	Carbon dioxide or Si–C stretching	2351	Carbon dioxide or Si–C stretching	− 2	–
2250	Weak S–H stretching “thiol”	2239	Weak C≡N stretching “nitrile” or weak C≡C stretching “alkyne”	11	Samson & Otaraku Ipeghan^74^
2169	Strong S–C≡N stretching “thiocyanate”	2167	Strong S–C≡N stretching “thiocyanate”	2	Dujearic-Stephane et al.^75^
1642	C=O amidic carbonyl or strong C=C stretching	1665	C=O, Carbonyl group “ketonic carbonyl” “conjugated ketone”	− 23	Di et al.^76^
1542	C=C stretching or Amide–II (protein N–H bend, C–N stretch)	1547	Strong N–O stretching “nitro compound”	− 5	Varotsis et al.^77^
1447	CH_2_–bending vibrations of the methyl group; lipids and proteins)	1456	Medium C–H binding “alkane”	− 9	Karar et al.^78^
		1450	Medium C–H binding “methyl group”	–	–
1390	Medium O–H binding	–	–	–	Li et al.^79^
1247	Medium C–N stretching “amine”	–	–	–	Bashir et al.^80^
1167	Strong C–O stretching	1152	C–O stretching	− 15	Aghazadeh & Nikpassand^81^
1106	Strong C–O stretching	–	–	–	Aghazadeh & Nikpassand^81^
1031	Strong S=O stretching	1030	Strong S=O stretching “sulfoxide”	1	Sangar et al.^82^
880	Strong C–H binding	–	–	–	Gopi et al.^83^
778	Strong C–H binding	768	Strong C–H binding	− 10	Gopi et al.^83^
731	Strong C–H binding	–	–	–	Gopi et al.^83^
669	Strong C–halogenated compound “Cl, Br or I”	676	Strong C–halogenated compound “Cl, Br or I”	− 7	Roeleveld et al.^84^
605	Phosphate	–	–	–	France et al.^85^
532	Two bi-phenyl rings “conjugated”	–	–	–	Ravi et al.^86^
456	Si–O stretching	461	Si–O binding	− 5	Ravi et al.^86^

The scale of FTIR spectra for the scanned samples is in the wave number range of 400–4000 cm^−1^. The FTIR spectrum of *Gelidium corneum* biomass before the biosorption process of Remazol brilliant blue dye and Hg^2+^ revealed the absorption bands at ν = 4069, 3442, 2934, 2349, 2250, 2169, 1642, 1542, 1447, 1390, 1247, 1167, 1106, 1031, 880, 778, 731, 669, 605, 532, and 456 cm^−1^. Besides, the FTIR spectrum of *Gelidium corneum* biomass after the biosorption process of both Remazol brilliant blue dye and Hg^2+^ revealed the same previous characteristic absorption bands with the absence of the absorption bands at ν = 1390, 1247, 1106, 880, 731, 605 and 532 cm^−1^. Similarly, an absorption band at ν = 1450 cm^−1^ characterized by C–H binding vibration appeared in the IR spectrum after the biosorption process. The shifts in the values of the absorption bands indicated the role of the functional groups in the biosorption process of both Remazol brilliant blue dye and Hg^2+^ from the aqueous solution.

The FTIR spectrum of the *Gelidium corneum* biomass before the bio-adsorption process of Remazol brilliant blue dye and Hg^2+^ revealed absorption bands characterized for various functional groups. Consequently, an absorption band at ν = 4069 cm^−1^ was identified for –OH hydroxyl group stretching as indicated by Bhattacharya et al.^71^. Li et al.^72^ established the absorption band at ν = 3442 cm^−1^ for –OH or N–H stretching vibration band. In addition, Zhao et al.^73^ characterized the absorption band at ν = 2934 cm^−1^ for C–H stretching of aliphatic hydrogens or OH stretching alcohol. The absorption band at ν = 2250 cm^−1^ is attributed to a weak S–H stretching “thiol” as reported by Samson and Otaraku Ipeghan^74^. On the other hand, Dujearic-Stephane et al.^75^, confirmed the absorption band at ν = 2169 cm^−1^ is related to a strong S–C≡N stretching vibration band “thiocyanate”. Moreover, the FTIR spectrum of the *Gelidium corneum* biomass before the bio-adsorption process shown a characteristic absorption band at ν = 1642 cm^−1^ owing to amidic carbonyl (C=O) or strong C=C stretching as reported by Di et al.^76^. The absorption band at ν = 1542 cm^−1^ is attributed to the C=C stretching or amide-II (protein N–H bend, or C–N stretch) Varotsis et al.^77^. Karar et al.^78^, have reported the absorption band related to CH_2_-bending vibrations of the methyl group; lipids and proteins at ν = 1447 cm^−1^. Once again, a medium O–H binding group appeared at 1390 as indicated by Li et al.^79^. Moreover, the absorption band at ν = 1247 cm^−1^ is attributed to the C–N stretching vibration for amines Bashir et al.^80^. It was reported that the absorption bands due to C–O stretching appeared at ν = 1167, and 1106 cm^−1^ as cited by Aghazadeh and Nikpassand^81^. Besides, the absorption bands at ν = 1031 (strong S=O stretching)^82^, 880 (strong C–H binding “1,2,4-trisubstituted”), 778 (strong C–H binding “1,2,3-trisubstituted”), 731 (strong C–H binding “1,2-disubstituted”)^83^, 669 (strong C–halogen “halogen = Cl, Br or I” “halogenated compound”)^84^, 605 (phosphate)^85^, 532 and 456 (two bi-phenyl rings “conjugated”)^86^.

The FTIR spectrum of the *Gelidium corneum* biomass after the biosorption process of Remazol brilliant blue dye and Hg^2+^ revealed the followed absorption bands in detailed at ν = 4056 (–OH hydroxyl groups stretching “alcohol”), 3597, 3578, 3557 (strong broad –OH stretching), 2940 (medium C–H stretching “alkane”), 2351 (carbon dioxide or Si–C stretching), 2239 (weak C≡N stretching “nitrile” or weak C≡C stretching “alkyne”), 2167 (strong S–C≡N stretching “thiocyanate”), 1665 (C=O, carbonyl group “ketonic carbonyl” “conjugated ketone”), 1547 (strong N–O stretching “nitro compound”), 1456 (medium C–H binding “alkane”), 1450 (medium C–H binding “methyl group”), stretching bands at 1152 due to the carbonyl group, 1030 (strong S=O stretching “sulfoxide”), 768 (strong C–H binding “1,2,3-trisubstituted”), 676 (strong C–halogen “halogen = Cl, Br or I” “halogenated compound”) and 461 cm^−1^ (Si–O group binding).

The FTIR spectrum of the sample before biosorption process of the Remazol brilliant blue dye and mercury ions revealed an absorption band at 456 cm^−1^ characterized for Si–O stretching vibration band, however the same absorption band was shifted after the biosorption process to 461 cm^−1^^87^. The values of –OH stretching bands at ν = 3442 cm^−1^ are shifted to slightly higher values after the dye and mercuric ions biosorption process to ν = 3557–3597 cm^−1^^25^. The absorption band at 1642 cm^−1^ in the IR spectrum of the sample before biosorption of the dye and mercury ions is characterized for amidic carbonyl group or strong C=C stretching vibration was shifted to higher value at ν = 1665 cm^−1^ that characterized for ketonic carbonyl group of conjugated ketones^88^. A slight change in the value of CH_2_–bending vibrations was noticed before and after the biosorption process from ν = 1447 to 1456 cm^−1^^89^. A strong absorption band at ν = 778 cm^−1^ was established for C–H binding vibration was shifted after the biosorption process of mercuric ions and the dye and recorded at ν = 768 cm^−1^^90^.

On the other hand, an absorption band at ν = 1450 cm^−1^ was characterized for C–H binding vibration was recorded in the IR spectrum of the sample after the biosorption process and was not recorded in the sample before the biosorption process^91^. In addition, the IR spectrum of *Gelidium corneum* biomass after biosorption of Remazol brilliant blue dye and mercury ions specified the absence of the absorption bands in the range of ν = 1247–1390 cm^−1^ indicating the interaction of the surface with the Remazol brilliant blue dye and mercury ions and the disappearance of the characteristic groups such as O–H binding and C–N stretching groups^92^. In this sequence, the absorption bands at ν = 880, 731, 605 and 532 cm^−1^ that are characterized for strong C–H binding vibrations, phosphate and conjugated biphenyl rings are disappeared after the biosorption process^93^.

The changes in the absorption bands indicating the interaction of these functional groups on *Gelidium corneum* surface with the Remazol brilliant blue dye and mercury ions during the biosorption process. The FITR spectra revealed that the alcohols, amines, ethers, alkyl substituent, aromatics, and phosphate groups played a significant role in the biosorption process of Remazol brilliant blue dye and mercury ions.

### Electron dispersive spectroscopy (EDS)

**Figure 9:**
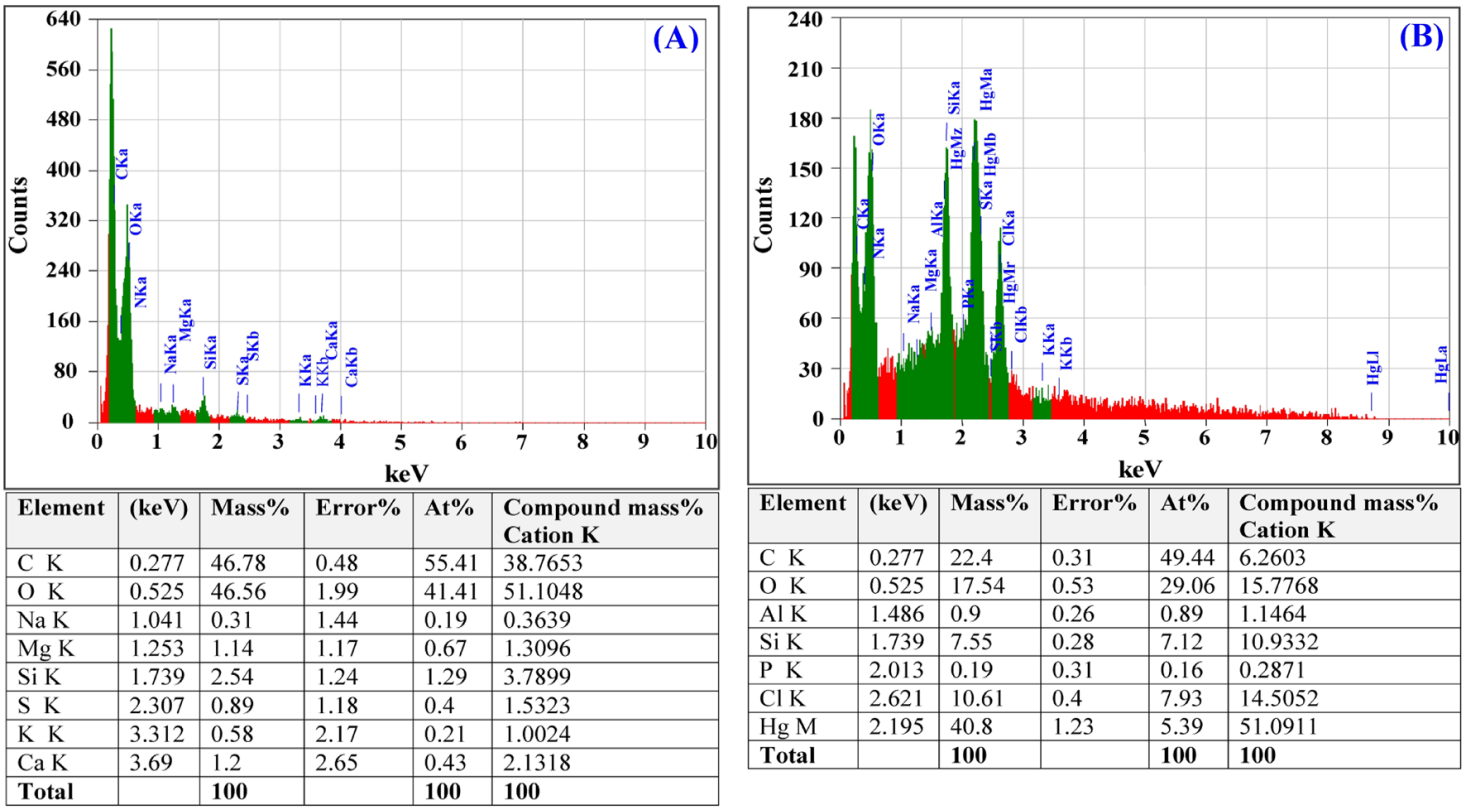
EDS analysis of *Gelidium corneum* biomass: (**A**) before and (**B**) after simultaneous biosorption of Hg^2+^ and Remazol brilliant blue dye.

EDS is a useful method used to characterize and to determine the elementary composition of biosorbents^94^. EDS analysis was used in this study to confirm the existence of Hg^2+^ on the surface of *Gelidium corneum* biomass cells after the biosorption process when compared to the cell surface before the biosorption process (Fig. 9A). The existence of an additional peak of Hg^2+^ attached to *Gelidium corneum* biomass cell surface after the biosorption process is revealed in the EDS spectrum (Fig. 9B), demonstrating the capacity of *Gelidium corneum* biomass to eliminate Hg^2+^ from aqueous solutions.

### Different mechanisms of the biosorption

**Figure 10:**
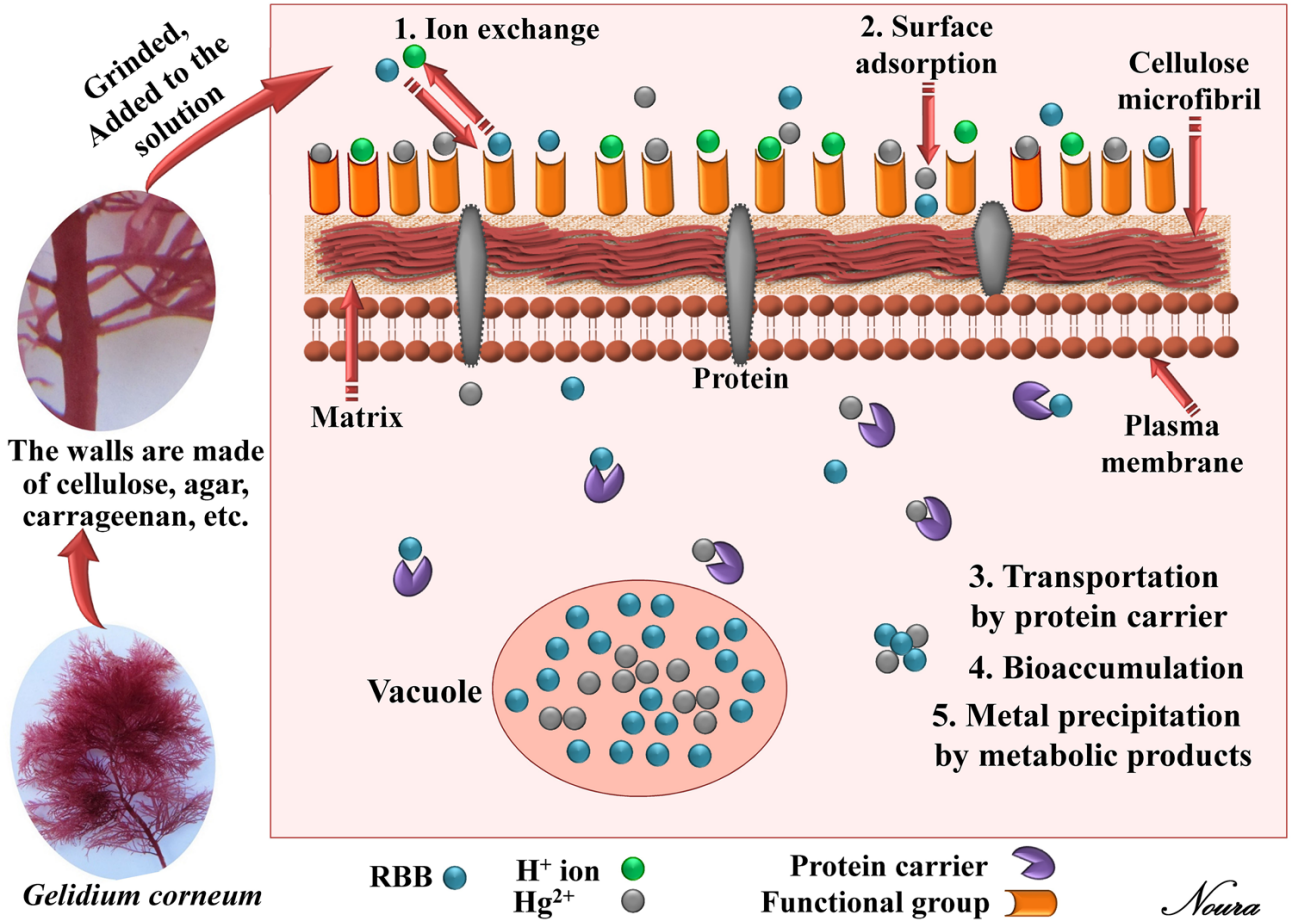
Schematic diagram showing the biosorption mechanisms of metal ions and dye by algal biomass.

The biosorption of heavy metals or dyes by algal biomass occurs primarily through cell wall interactions and involves a number of mechanisms (Fig. 10) that differ qualitatively and quantitatively depending on the species used, the source of the biomass, and the biomass's processing procedure. The mechanisms of the biosorption process by the marine algae biomass include binding to intracellular components and proteins, bioaccumulation within the cells, chelation, diffusion interior the cells or surface precipitation, complex formation between contaminants' cations and ligands on the algal surface, ion exchange^19^ and reduction reactions, accompanied by metallic precipitation on the cell wall matrix^95^. Ion-exchange is a vital concept in the biosorption process that takes place between various ions and protons at the biomass binding sites.

El-Naggar et al.^21^ recorded that the mechanism of the biosorption process is mainly dependent on the biomacromolecules on the algal surface. The walls of the red algae chemical composition are composed of microfibrillar cellulosic layer, xylan or mannan fibrils and an extensive matrix composed of various types of long-chained polysaccharides including agar, carrageenan, sulfated polygalactans, gelans (mucusy sugars) and proteins together with minerals. Some red algae species can absorb calcium from seawater and deposit calcium carbonate in their cell walls^34^. The cell wall components of the red algae, in addition to calcium carbonate contain a variety of functional groups on the surface of the algal biomass such as CH_2_, C–H, C=O, N–O, C–N, –OH, PO_4_, and –NH_2_. These groups can serve as adsorption sites that are responsible for metal ions and dye biosorption. Dulla et al.^20^ documented that the algae's biosorption capacity has been influenced by the presence of functional groups in their cell walls like sulphate, carboxylic, amino, and hydroxyl. These groups can be used to serve as binding sites for metal ions via a range of mechanisms that include ion exchange, electrostatic forces, and complexation.

## Materials and methods

### Collection and preparation of *Gelidium corneum* biomass as biosorbent

During the summer of 2018, the *Gelidium corneum* biomass was harvested from the Mediterranean Sea coast of Abu-Qir, Alexandria, Egypt. Alga was collected according to institutional, national, and international guidelines and legislation. To remove salts, sand, and other external impurities, the alga was thoroughly washed with running tap water. Identification was performed as described by Taylor^96^. After that, the washed algal biomass was dried at room temperature until it reached a constant weight and ground. The fine particles with an average size of 0.3–0.5 mm were selected and preserved in a dry place for future use.

### Preparations of Remazol brilliant blue dye and Hg^2+^ stock solutions

Stock solutions of both Remazol brilliant blue dye (Sigma-Aldrich) and Mercury (II) chloride (HgCl_2_); ACS reagent, ≥ 99.5%) were prepared. Remazol brilliant blue dye stock solution was made by dissolving weighted Remazol brilliant blue dye in distilled water to a concentration of 100 mg/L. By diluting Remazol brilliant blue dye stock solution, the desired work concentrations were developed. Mercury (II) chloride (HgCl_2_) stock solution is made by dissolving weighted HgCl_2_ in distilled water at a concentration of Hg^2+^, 300 mg/L. By diluting Mercury stock solution, the desired work concentrations were developed.

### The biosorption experiments

To investigate the effect of Hg^2+^ concentration, Remazol brilliant blue concentration, pH, contact time, and *Gelidium corneum* biomass concentration on the biosorption process, the biosorption experiments were carried out in a batch system in 250 mL Erlenmeyer flasks with a 100 mL working volume. Dry alga biomass has been extensively mixed with Remazol brilliant blue and Hg^2+^ solutions with different concentration levels, as seen in the 50 trials (Table 1). The initial pH was calibrated for each solution with a supplement of 0.1 N NaOH or 0.1 N HCl. For the duration of contact, the suspensions were incubated and agitated in a shaker incubator at 150 rpm and 30 °C.

### Optimization of the simultaneous biosorption of Hg^2+^ and a Remazol brilliant blue dye by FCCCD (face-centered central composite design)

FCCCD of 50 experimental runs, 10 axial points, 32 factorial and 8 replicates at the midpoints was used to optimize the five independent variables (initial pH level, contact time, Hg^2+^, *Gelidium corneum* biomass and Remazol brilliant blue dye concentrations) to maximize bioremoval of Remazol brilliant blue dye and Hg^2+^ from the binary solution. These independent variables vary on three coded levels (− 1, 0 and + 1) (Table 1). Furthermore, the linear, quadratic, and mutual interactions effects among the selected process variables on the simultaneous bioremoval of Hg^2+^ and Remazol brilliant blue dye should be investigated. In each trial, the average of the triplicate responses (Y) for Remazol brilliant blue dye removal (percent) and Hg^2+^ removal (percent) was used. To save resources, all the experiments were done at 30 °C.

The following equation of second-degree polynomial was used to evaluate the correlations between the selected tested variables and the responses (Hg^2+^ and Remazol brilliant blue dye biosorption percentages):

3 $$Y = \beta_{0} + \sum\limits_{i} {\beta_{i} X_{i} } + \sum\limits_{ii} {\beta_{ii} X_{i}^{2} } + \sum\limits_{ij} {\beta_{ij} X_{i} X_{j} }$$

In which Y is the predicted Hg^2+^ or Remazol brilliant blue dye biosorption percentages, X_i_ is the coded variables levels, β_ij_ (interaction coefficients), β_i_ (linear coefficient), β_0_ (regression coefficients), β_i_ (linear coefficient), and β_i_ (linear coefficient).

### Analytical methods

The contents of every flask of FCCCD experiment have been centrifuged at 6000 × *g*.

The supernatants were analyzed for determination of the residual concentration of Hg^2+^ of each FCCCD experiment using Atomic Absorption Spectroscopy (AAS, Buck scientific 2 hydrous system series Atomic Absorption (USA) by air acetylene system) at the Biotechnology Unit, Mansoura University, Egypt according to “standard methods for the examination of water and wastewater 23rd edition 2017”^97^. The residual Remazol brilliant blue of each FCCCD experiment was measured using a UV/Vis spectrophotometer at the wavelength of highest absorbance (λ_max_ 500).

The following formula was used to calculate the Remazol brilliant blue and Hg^2+^removal percentages:

4 $${\text{Removal}}\;{\text{efficiency}}\left( \% \right) = \frac{{{\text{C}}_{{\text{i}}} - {\text{C}}_{{\text{f}}} }}{{{\text{C}}_{{\text{i}}} }} \times 100$$

where C_i_, C_f_ are the initial and residual Remazol brilliant blue dye concentrations or Hg^2+^ concentrations; respectively.

### Statistical analysis

For the experimental design, statistical analysis and drawing of three-dimensional plots of the area, STATISTICA version 8 and Design Expert version 7 Windows software were applied.

### Fourier-transform infrared (FTIR) spectroscopy

The dehydrated *Gelidium corneum* biomass samples were subjected to FTIR analyses in order to determine the functional groups on the algal surface (chemical characteristics) that may be important to the binding of Hg^2+^ and Remazol brilliant blue dye to the biomass. *Gelidium corneum* biomass samples (before and after Remazol brilliant blue dye and Hg^2+^ biosorption) were blended with KBr pellets and analyzed with the FTIR spectroscopy “Thermo Fisher Nicolete IS10, USA spectrophotometer” within the range of 400–4000 cm^−1^.

### Scanning electron microscopy (SEM) analysis

To investigate the algal cell surface and to evaluate the biosorption of Hg^2+^ and Remazol brilliant blue dye, the dehydrated *Gelidium corneum* biomass samples have been gold-coated and examined with SEM before and after biosorption of Remazol brilliant blue dye and Hg^2+^.

### Energy dispersive X-ray spectroscopy (EDS)

to determine the chemical composition of *Gelidium corneum* biomass samples before and after biosorption of Hg^2+^ and Remazol brilliant blue dye, the dehydrated samples were examined using TEM/EDS.

## Conclusion

The use of *Gelidium corneum* biomass for the biosorption of Hg^2+^ ions and Remazol brilliant blue dye has been identified as a potential and practical alternative to traditional remediation methods. Hg^2+^ was removed at a rate of 97.09 percent and Remazol brilliant blue dye was removed at a rate of 88.67 percent using the optimum predictable conditions that are initial Remazol brilliant blue dye concentration of 99.33 mg/L, initial Hg^2+^ concentration of 216.87 mg/L, initial pH level of 4.85, contact time of 210.34 min and the algal biomass concentration of 3.08 g/L. The biomass of *Gelidium corneum* could be promising and offer the merits of competent application due to their potential properties and high metal binding capacities due to the presence of various functional groups in their cell walls, including alcohols, amines, ethers, alkyl substituents, aromatics, and phosphate groups, which serve as adsorption sites and played a significant role in the biosorption process of Remazol brilliant blue dye and mercury ions. The present investigation highlighted the simultaneous removal of dyes and heavy metal ions and successfully employed the biomass of *Gelidium corneum* for the biosorption of Hg^2+^ ions and Remazol brilliant blue dye from a binary system. However, further research is needed to study kinetics, isotherms and thermodynamics models, in addition to biomass reusability.
